# Graph-based exploitation of gene ontology using GOxploreR for scrutinizing biological significance

**DOI:** 10.1038/s41598-020-73326-3

**Published:** 2020-10-07

**Authors:** Kalifa Manjang, Shailesh Tripathi, Olli Yli-Harja, Matthias Dehmer, Frank Emmert-Streib

**Affiliations:** 1 Predictive Society and Data Analytics Lab, Tampere University, Tampere, Korkeakoulunkatu 10, 33720 Tampere, Finland; 2 Computational Systems Biology, Tampere University, Tampere, Korkeakoulunkatu 10, 33720 Tampere, Finland; 3 Institute for Systems Biology, Seattle, WA USA; 4 Department of Biomedical Computer Science and Mechatronics, UMIT-The Health and Life Science University, 6060 Hall in Tyrol, Austria; 5 College of Artificial Intelligence, Nankai University, Tianjin, 300350 China; 6 Institute of Biosciences and Medical Technology, Tampere University, Tampere, Korkeakoulunkatu 10, 33720 Tampere, Finland

**Keywords:** Computational models, Data processing, Gene ontology, Computational science, Computer science, Scientific data, Software, Statistics

## Abstract

Gene ontology (GO) is an eminent knowledge base frequently used for providing biological interpretations for the analysis of genes or gene sets from biological, medical and clinical problems. Unfortunately, the interpretation of such results is challenging due to the large number of GO terms, their hierarchical and connected organization as directed acyclic graphs (DAGs) and the lack of tools allowing to exploit this structural information explicitly. For this reason, we developed the R package GOxploreR. The main features of GOxploreR are (I) easy and direct access to structural features of GO, (II) structure-based ranking of GO-terms, (III) mapping to reduced GO-DAGs including visualization capabilities and (IV) prioritizing of GO-terms. The underlying idea of GOxploreR is to exploit a graph-theoretical perspective of GO as manifested by its DAG-structure and the containing hierarchy levels for cumulating semantic information. That means all these features enhance the utilization of structural information of GO and complement existing analysis tools. Overall, GOxploreR provides exploratory as well as confirmatory tools for complementing any kind of analysis resulting in a list of GO-terms, e.g., from differentially expressed genes or gene sets, GWAS or biomarkers. Our R package GOxploreR is freely available from CRAN.

## Introduction

The gene ontology (GO) consortium funded by the National Institute of Health (NIH) started in 1998. Initially, GO contained only three model organisms but extended since then to over 3200^1,2^. The ontology is structured into three distinct aspects of gene function, namely, molecular function (MF), cellular component (CC), and biological process (BP) together with over 45, 000 terms and 130, 000 relations. However, the majority of information is centered around ten model organisms (human, mouse, rat, zebrafish, drosophila, *C. elegans*, *D. discoideum*, *S. cerevisiae*, *S. pombe*, *A. thalia* and *E. coli*)^2^. In addition, GO includes annotations by linking specific gene products to GO-terms. This allows the connection between genes and GO-terms for deriving organism-specific information. Currently, GO is the most comprehensive and widely used knowledge base concerning functional information about genes^3–6^.

A reason for the widespread applicability of GO is its generality. That means instead of providing solutions to particular problems, GO provides generic information that can be connected to any list of genes or gene products regardless of the type of upstream analysis that generated such a list. For instance, investigations that can lead to a list of genes are from studies about differentially expressed genes or gene sets, GWAS (genome-wide association study), biomarkers or gene regulatory networks^7–13^. These studies could be of biological, medical, clinical or pharmacological nature making GO useful across the life and health sciences.

Interestingly, despite the widespread usage of GO for a number of different application types^7,14,15^, for exploring the GO knowledge base from a graph theoretical perspective^16,17^ the available tools are surprisingly sparse and only very basic functions are available for obtaining structural information^18–20^. However, no dedicated functions are ready-for-use that give us, e.g., information about the GO-level of a GO-term, the category (regular node, jump node or leaf node) of a GO-term, the adjacency matrix of the GO-DAG of BP terms or all GO-terms on a specific GO-level, to name just a few. Furthermore, existing tools do not provide means for reducing the overall complexity of GO that would be amenable, for instance, for a visualization. Given the size of GO containing thousands of GO-terms, such a simplification would be highly desirable.

For these reasons, we created the R package GOxploreR to fill this gap. Our package provides direct access to structural information allowing the efficient exploitation of graph-theoretical properties of a DAG (directed acyclic graph) for further analysis. We provide also information on a low level. For instance, given a list of Entrez Gene IDs our package includes an (online) function to provide the BP, MF or CC of GO-terms associated with these genes. To retrieve the most current GO-terms, we use the biomartR package to query the Ensembl website. However, for obtaining fast information, we added also an offline version of these functions with pre-assembled information. This functionality is supported for ten organisms.

Aside from functions for the quantification of structural properties of GO-DAGs, we provide also visualization capabilities. Due to the size of GO our visualizations aim at a simplified representation. Specifically, by categorizing GO-terms into three classes—called regular nodes (RN), jump nodes (JN) and leaf nodes (LN)—we obtain a simplified representation of a GO-DAG with at most three nodes on each GO-level and the connections among them. These categories simplify the semantic attributes of GO-terms significantly yet provide important information regarding their connectivity. In this way, the GO-DAG of human for BP with 29, 699 GO-terms is reduced to a simplified DAG with 39 nodes, which is amenable for a visualization. We provide also extensions of such a visualization by, e.g., filtering for a set of GO-terms. This leads to a further reductions of complexity and can be utilized for compact visualizations of large lists of significant genes, gene sets or pathways. Finally, we provide a function for prioritizing a list of GO-terms as obtained, e.g., from differentially expressed genes, that reflects the structural positions of these GO-terms and their biological-semantic importance within the entire GO-DAG.

In general, one of the main applications of GO is the identification of over- or under-represented GO-terms for a specified gene list (as a result, e.g., from identifying differentially expressed genes) utilizing a hypergeometric test (also known as Fisher’s exact test)^21,22^. A problem with this is that GO has a hierarchical structure in the form of a directed acyclic graph (DAG), which means that the GO-terms are dependent on each other. However, the above approaches ignore this dependency structure. For compensating this omission, semantic measures have been suggested, e.g., utilizing frequencies to assess the similarity/distance between GO-terms^23^. Alternatively, information about the connection of GO-terms has been included to a certain degree for enrichment analysis, e.g.,^24^. Although such approaches are more informative, in practice, they are often ignored and the structure-less methods are preferred because they are simpler to apply and interpret. Another problem is that different semantic measures seem to be preferable for particular biological data and applications, which further complicates the selection of such measures enormously^25^.

In contrast, the R package GOxploreR is different to the above approaches in the following way. Specifically its main features include (I) a direct access to structural features of GO, (II) a structure-based ranking of GO-terms, (III) a mapping from a GO-DAG to a reduced GO-DAG, (IV) a visualization of reducuded GO-DAGs and (V) an algorithm for prioritizing GO-terms. That means the providesd features are meant to complement, e.g., approaches for identifying enriched GO-terms by providing alternative approaches for the analysis of GO-terms. Overall, GOxploreR can help in improving some of the above discussed shortcomings by providing novel ways for graph-based exploitations of the GO knowledge base to simplify the interpretation of large sets of significant GO-terms by utilizing structural information from the underlying DAG. Due to the fact that such a list of GO-terms can come from any type of upstream analysis, GOxploreR is a very versatile and flexible tool with respect to potential applications in the life and health sciences.

This paper is organized as follows. In the next section, we describe the underlying methodology of GOxploreR and the provided functionality. Then we showcase the applicability of GOxploreR by highlighting some of its features and implemented functions. This paper finishes with a discussion of the available functions, a comparison to existing tools and concluding remarks.

## Methods

In this section, we provide technical information about the main features provided by GOxploreR. First, we discuss how one obtains a directed acyclic graph (DAG) for given GO-terms. Then we discuss organism-specific GO-DAGs and a mapping that converts such a DAG into a reduced GO-DAG. Finally, we discuss an algorithm for prioritizing GO-terms.

### Determining the GO-DAG

**Figure 1:**
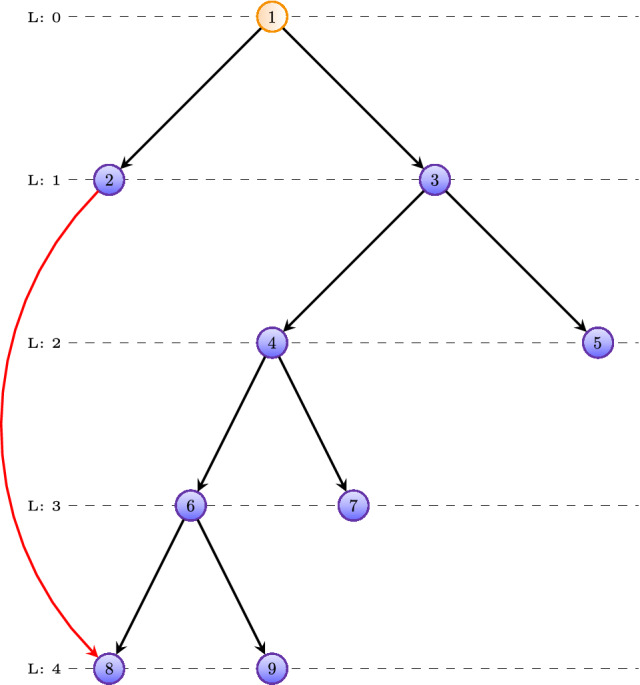
An example for a toy GO-DAG containing 9 GO-terms, whereas each node corresponds to one GO-term. The children of a node can jump over levels, as shown in red for the connection between node 2 and 8.

The problem with existing packages is that none provides a function to directly obtain a GO-DAG for a domain, i.e., BP, MF or CC, in the form of an adjacency matrix. Instead, they provide local information which needs to be used for *deducing* such a tree tediously. For instance, GOdb provides the function GOBPCHILDREN to get the children of a GO term for BP. For the other two domains similar functions are available. The problem is that a children node does not need to be on the next hierarchy level but can jump further down the DAG. For an example see Fig. 1. In this figure, the child of node 2 is node 8 which is located on level 4, i.e., the child jumps from level 1, the location of its parent, to level 4.

The following example demonstrates how one can deduce a GO-DAG from this information. First, we list all children of a GO term (as obtained via the command GOBPCHILDREN).

1 $$\begin{aligned} CH(x_1)&= \{x_2, x_3\} \end{aligned}$$

2 $$\begin{aligned} CH(x_2)&= \{x_8\} \end{aligned}$$

3 $$\begin{aligned} CH(x_3)&= \{x_4, x_5\} \end{aligned}$$

4 $$\begin{aligned} CH(x_4)&= \{x_6, x_7\} \end{aligned}$$

5 $$\begin{aligned} CH(x_5)&= \emptyset \end{aligned}$$

6 $$\begin{aligned} CH(x_6)&= \{x_8, x_9\} \end{aligned}$$

7 $$\begin{aligned} CH(x_7)&= \emptyset \end{aligned}$$

8 $$\begin{aligned} CH(x_8)&= \emptyset \end{aligned}$$

9 $$\begin{aligned} CH(x_9)&= \emptyset \end{aligned}$$

The root node is unique and we assign it the level 0, i.e., $L(x_1)=0$. The children for the root node receive as first assignment for a level the value $L(x_1) + 1=1$, i.e.,

10 $$\begin{aligned} L(x_2)&= \{1\} \end{aligned}$$

11 $$\begin{aligned} L(x_3)&= \{1\} \end{aligned}$$

We wrote the right-hand side as a set because if such a node appeares again, we just add the new level value to this set. Going through the list of children, we assign each children of a node $x_i$ the value $L(x_i) + 1$.

12 $$\begin{aligned} CH(x_2) \rightarrow L(x_8)&= \{2\} \end{aligned}$$

13 $$\begin{aligned} CH(x_3) \rightarrow L(x_4)&=\{2\}, \, L(x_5) =\{2\} \end{aligned}$$

14 $$\begin{aligned} CH(x_4) \rightarrow L(x_6)&=\{3\}, \, L(x_7) =\{3\} \end{aligned}$$

15 $$\begin{aligned} CH(x_6) \rightarrow L(x_8)&=\{2,4\}, \, L(x_9) =\{4\} \end{aligned}$$

From the last line we see that $x_8$ appears once on level 2 and once on level 4, which is correct if one looks at Fig. 1. However, there is just one correct level for $x_8$ and this is level 4. In general, if more than one level is assigned to a node then the correct one is the largest of these values.

Such a GO-DAG can be constructed for every domain, i.e., biological process, molecular function and cellular component. In our package, we call the resulting graphs:

- g.GO-DAG.BP: A DAG for all GO-terms of biological processes.
- g.GO-DAG.MF: A DAG for all GO-terms of molecular functions.
- g.GO-DAG.CC: A DAG for all GO-terms of cellular components.

### Organism-specific GO-DAG

Starting from a GO-DAG for a domain, as constructed in the previous section and using a list of all genes from an organisms, we can map these genes to GO-terms. For a particular organism, not all GO-terms may be present but only a subset. Such a subset can then be mapped back to the entire GO-DAG of the knowledge base. This gives a subtree of the general GO-DAG that is organism-specific. Using the function GetDAG(organism = o.name, domain = "BP") one obtains, e.g., a GO-DAG of BPs for the organism given by ’o.name’. For all domains, the following functions can be used:

- GetDAG(organism = o.name, domain = "BP"): A sub-DAG for all GO-terms of biological processes for organism ’o.name’.
- GetDAG(organism = o.name, domain = "MF"): A sub-DAG for all GO-terms of molecular functions for organism ’o.name’.
- GetDAG(organism = o.name, domain = "CC"): A sub-DAG for all GO-terms of cellular components for organism ’o.name’.

### Reduced GO-DAG

Visualizing one of the GO-DAGs determined above (for all GO-terms or for organism-specific GO-terms) is usually challenging because of the size of such graphs containing thousands of GO-terms corresponding to nodes in a graph. For this reason, we derive a simplified GO-DAG, containing only dozens of nodes, that can be easily visualized to obtain a global overview of all used GO-terms.

In order to simplify a GO-DAG, we introduce the following categorization of GO-terms, excluding the root node. This categorization is applied to each level separately:

- A GO-term is in category ’leaf node’ (LN) if it has no children.
- A GO-term is in category ’regular node’ (RN) if all its children are on the next level.
- A GO-term is in category ’jump node’ (JN) if it has children and at least one of these is not on the next level.

We apply this categorization for all GO-terms. This results in the mapping

- GO-term *X* $\rightarrow$ GO-term category on level L

That means we have functions of the form

16 $$\begin{aligned} (c, l) = f(X) \end{aligned}$$

with $c \in \{$LN, RN, JN$\}$ and $l \in \mathbb {N}$. For instance, from Fig. 1 follows $3 \rightarrow$ RN on level 1 and $2 \rightarrow$ JN on level 1, which can be written formally as

17 $$\begin{aligned} (\text{ RN }, 1)&= f(3) \end{aligned}$$

18 $$\begin{aligned} (\text{ JN }, 1)&= f(2) \end{aligned}$$

Algorithmically, the implementation is described in 1.

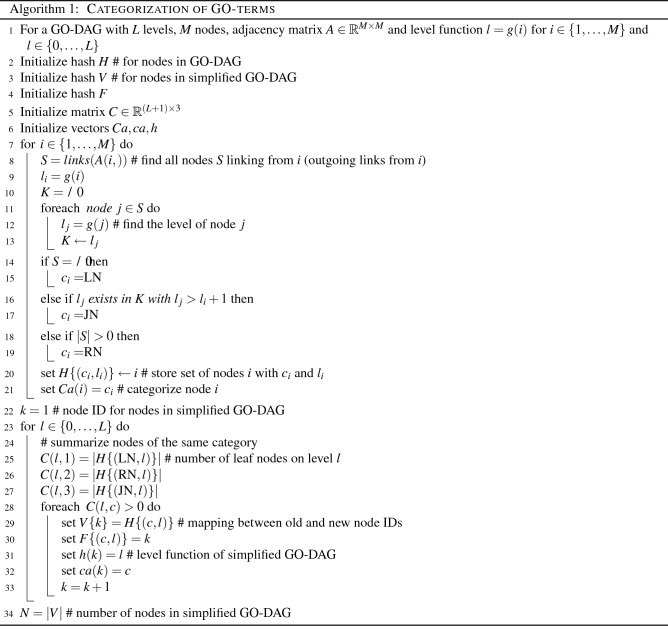



In addition to the node categorization, we need to find the connections between these nodes. This is realized via the implementation shown in Algorithm 2.

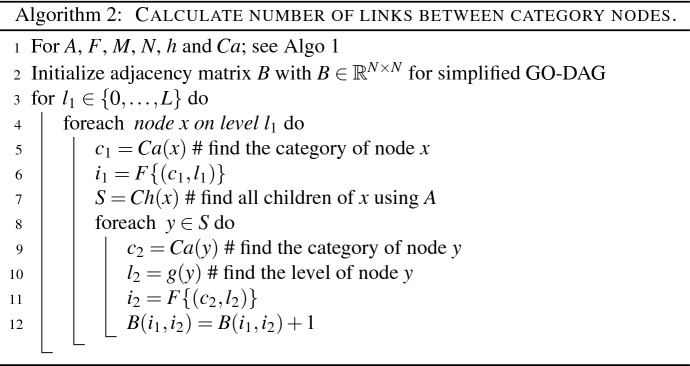



Overall, a GO-DAG is described by an adjacency matrix *A* and a level function *g* and analogously, a reduced GO-DAG is described by adjacency matrix *B* and level function *h* and *C* (number of original nodes summarized by a new category).

**Figure 2:**
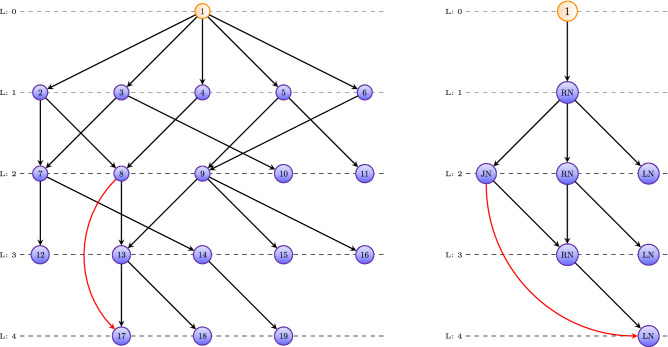
An example for the construction of a reduced GO-DAG. Left: An ordinary GO-DAG with 19 GO terms is shown. Right: The reduced GO-DAG with 8 nodes summarizes the left graph. Note, the nodes in the right graph are no GO-terms but node categories, i.e., either RN, JN or LN.

In Fig. 2 we show a complete example for this mapping. The GO-DAG on the left-hand side has 19 GO terms and the resulting simplified GO-DAG on the right-hand side has only 8 nodes, whereas these nodes correspond to the three GO categories (RN, JN & LN) defined above. As one can see, each level will contain at most 3 nodes because this is the number of different categories. However, it is possible to have even fewer nodes, if a category is absent on a level.

Importantly, this transformation can be applied to any GO-DAG, regardless if this DAG is for all GO terms of, e.g., BPs, or for an organism-specific GO-DAG.

### Prioritizing lists of GO-terms

In general, the comparison of GO-terms with respect to their biological-semantic importance is complex. However, the comparison of GO-terms along a path is much simpler because the higher a level of a GO-term is the more specific is its biological information^26^. That means *vertically* one wants to traverse a DAG along a path as far down as possible. This implies that the GO-term at the end of a path is most interesting compared to all other GO-terms along this path. This increase in the semantic meaning along *vertical* paths is exploited by our algorithm for prioritizing lists of GO-terms.

**Figure 3:**
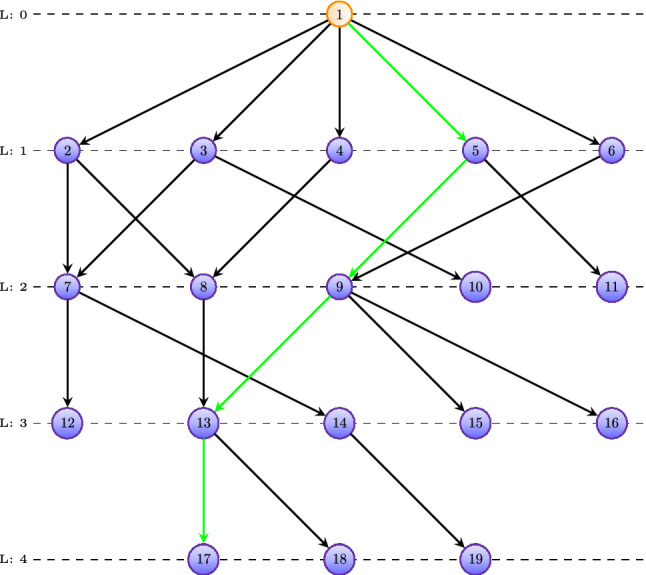
Shown is a path (green) in a GO-DAG, where nodes correspond to GO-terms. Along this path, the biological semantics increases from node to node the further down one traverses the path.

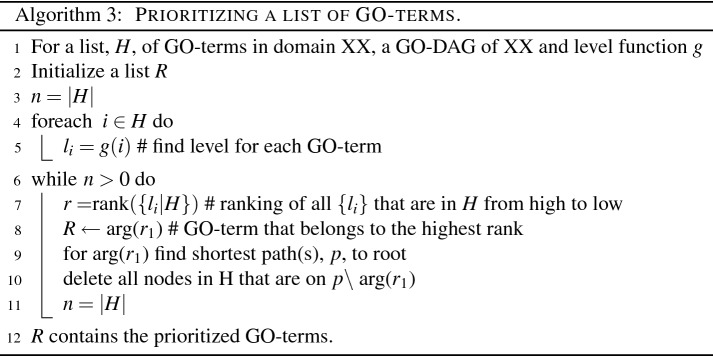



Our algorithm applies the above described logic iteratively, by starting from the GO-term at the highest level and searches all shortest paths to the root node. Then all GO-terms along these shortest paths are removed from the list and the procedure starts over. See Fig. 3 for a visualization. In this figure, one shortest path from node 17 to the root node is shown. The pseudo-code of this is shown in Algorithm 3. Here XX corresponds to BP, MF or CC. The algorithm guarantees that for a non-empty list, *H*, of GO-terms the resulting set, *R*, containing the prioritized GO-terms consists of at least one GO-term. For instance, say $H=\{5, 9, 17\}$. Then our algorithm starts at node 17 and searches all shortest paths to the root. One of these is highlighted in green in Fig. 3. As a result, the nodes 5 and 9 are eliminated because they appear on a lower hierarchy level than node 17. In this case, the final result of our algorithm gives $R=\{17\}$.

Overall, our prioritizing algorithm provides a parameter- and assumption-free, non-redundant ranking of GO-terms that exploits only vertical structural information of GO.

### Technical details about GO

For the construction of the various DAGs, we are only utilizing information from GO-basic. The information about this can be obtained from the go-basic.obo file, which can be obtained from the Gene Ontology website (http://geneontology.org/docs/download-ontology/). This file contains the basic version of GO and it is guaranteed that the resulting DAG is acyclic and annotations can be propagated through the graph. We would like to note that the relations included in this, i.e., "is_a", "part_of", regulates, "negatively_regulates" and "positively_regulates" also guarantee transitivity (NB: transitivity is not obeyed by "has_part" relations which are included in GO-core available from the *go.obo* file via the GeneOntology website).

## Results

In the following sections, we highlight some of the features provided by the GOxploreR package and show some example applications.

### Structural exploration of GO

In Table 1, we show an overview of the organisms supported by the GOxploreR package. Overall, at the moment ten organisms are supported corresponding also to the main organisms within the GO database. The second column in Table 1 shows the option name as used for arguments in functions.

**Table 1:** An overview of the organisms supported by the GOxploreR package.

**Organism**	Option name	Genes	Levels	BP-terms
Human	"*Homo sapiens*"/"Human"	19155	19	12436
Mouse	"*Mus musculus*"/"Mouse"	20929	18	12328
Caenorhabditis elegans	"*Caenorhabditis elegans*"/"Worm"	14697	17	3689
Drosophila melanogaster	"*Drosophila melanogaster*"/"Fruit fly"	12683	18	5323
Rat	"*Rattus norvegicus*"/"Rat"	19383	18	11584
Baker’s yeast	"*Saccharomyces cerevisiae*"/"Yeast"	5502	17	3050
Zebrafish	"*Danio rerio*"/"Zebrafish"	20718	18	5404
Arabidopsis thaliana	"*Arabidopsis thaliana*"/"Cress"	25891	17	4059
S. pombe	"*Schizosaccharomyces pombe*"/"Fission yeast"	5055	16	2973
Escherichia coli	"*Escherichia coli*"/"*E.coli*"	3449	15	1491

For instance, the following command gives for the gene list ’c(10212, 9833)’ containing Entrezgene IDs information about the associated GO-terms and hierarchy levels.

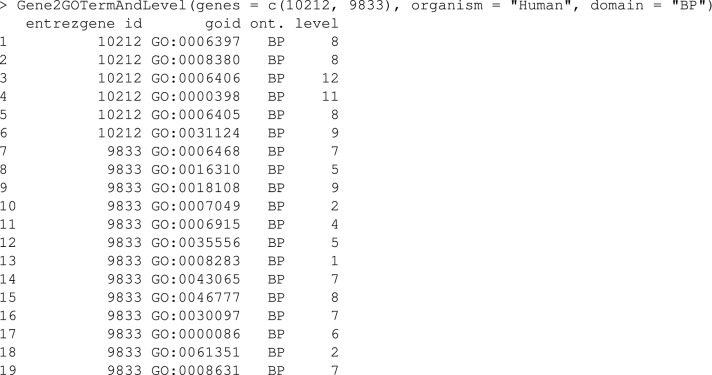



In case a list of GO-terms is already available the corresponding hierarchy levels can be obtained with the command ’GOTermXXOnLevel’. Here ’XX’ is either BP, MF or CC. In the following, ’XX’ corresponds always to one of these three domains.

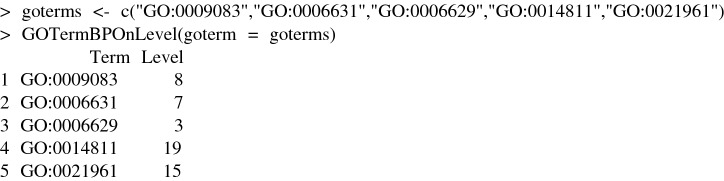



For the analysis of enriched GO-terms, one frequently wants to limit such an analysis to more informative GO-terms which are located toward higher hierarchy levels. In order to obtain all GO-terms located on a specific hierarchy level one can use the function ’Level2GOTermXX’.

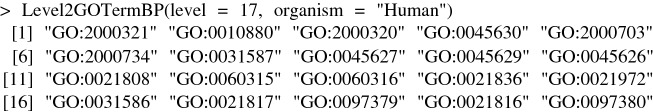



**Figure 4:**
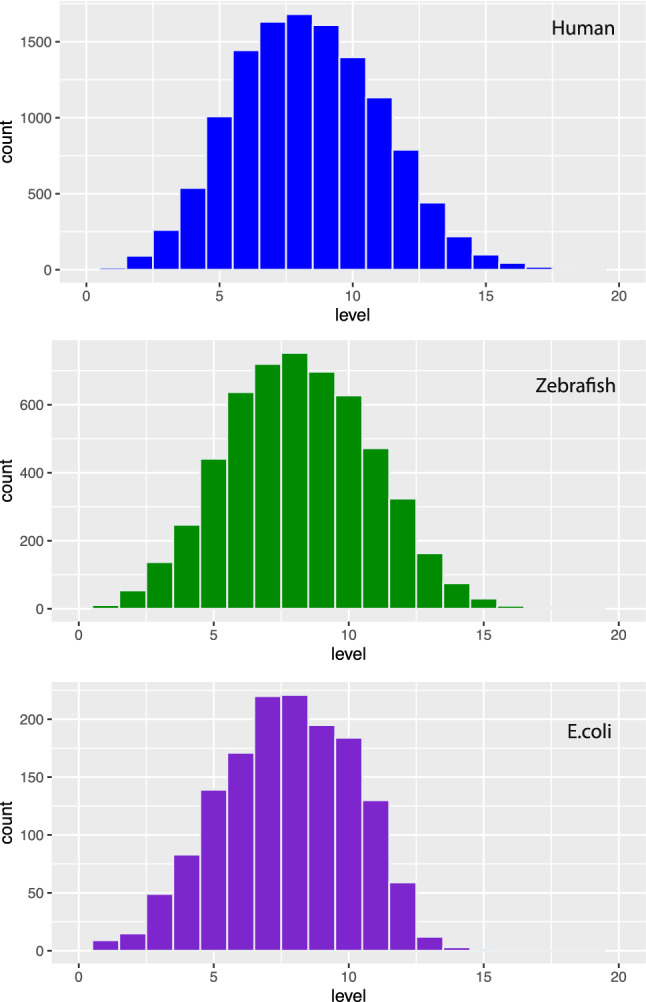
Distribution of GO-terms of BP for human (top), zebrafish (middle) and *E. coli* (bottom). The x-axis corresponds to the hierarchy level of the corresponding GO-DAG.

It is interesting to highlight that the children of a GO-term in a GO-DAG can ’jump’ to different levels. For instance, using the function ’GOTermXX2ChildLevel’ gives the GO-terms as well as the corresponding hierarchy levels of these.

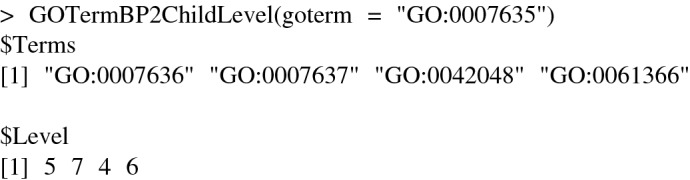



Here the GO-term "GO:0007635" is on level 3, however, its children are not only on level 4. The reason for this is that in GO there are no cross links on the same level. That means the children of any GO-term are always on a lower level because the terms are more specific. This implies that "GO:0007636" which is located on level 5 has (at least one) parent node located on level 4. In order to find this parent(s) we can use the following.

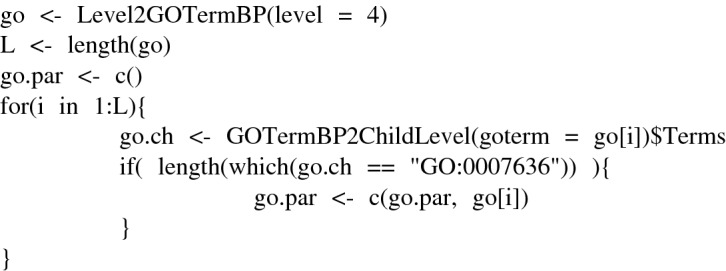



In this case there are 1166 GO-terms on level 4 and the only parent of "GO:0007636" is "GO:0007630".

It is important to note that GO does not only provide one DAG but several different ones. The reason for this is that each organism has a specific number of genes, and from these genes one obtains only a subset of all GO-terms that are connected to an organism. In total there are eleven GO-DAGs available from GOxploreR, ten for the organisms and one for all GO-terms.

In order to demonstrate the differences in the GO-terms for different organisms, we show in Fig. 4 the distribution of GO-terms of BP for human (top), zebrafish (middle) and *E. coli* (bottom). The x-axis corresponds to the hierarchy level of the corresponding GO-DAG of BP. As one can see for human one has a GO-DAG with 19 hierarchy levels whereas for zebrafish one has 16 and for *E. coli* 14. Furthermore, also the number of GO-terms on these levels is considerably different from each other as can be seen from the counts (number of GO-terms) on the y-axis. In Table 1, we show an overview of the number of levels (column four) and the number of GO-terms of BP (column five) for all ten organisms. For completeness, we want to mention that if one does not specify the organism in the command ’Level2GOTermBP’ one can obtain a total number of 29698 GO-terms of BP for all levels.

### Structure-based ranking of GO-terms

Maybe the most popular application of GO is the identification of enriched GO-terms for a list of genes. Unfortunately, as a result from such an analysis it is not uncommon to find large numbers of GO-terms making a focused discussion very difficult. However, a GO-DAG provides information that can be utilized for an exploratory analysis of such a list. Specifically, the hierarchy levels of GO-terms can be utilized. Despite the fact that a GO-level is not an absolute indicator for biological specificity it provides still valuable information^26^. Using our function *GOTermBPOnLevel* gives the GO-levels of BP for a list of GO-terms allowing, e.g., a simple ordering for complementing an enrichment analysis.

**Figure 5:**
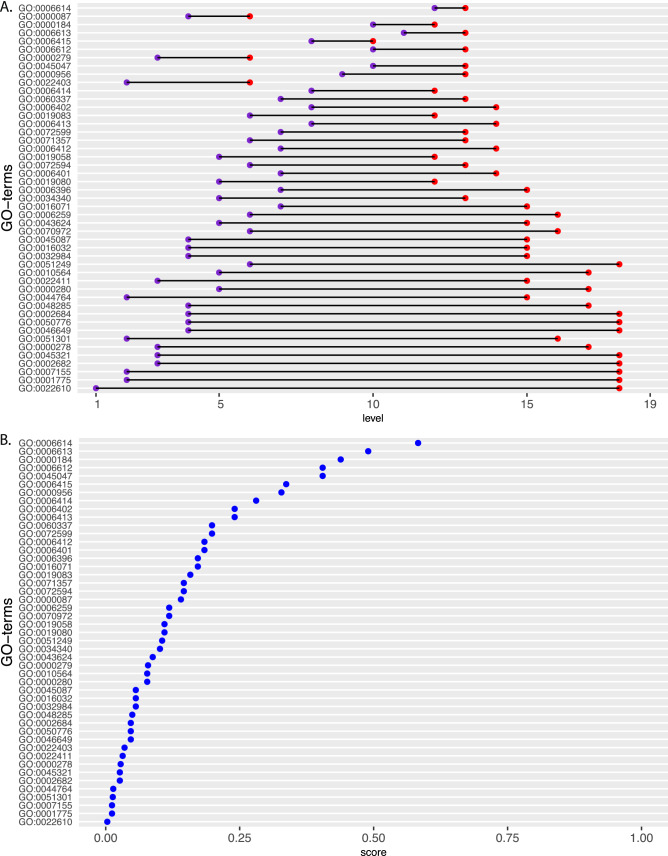
(**A**) The hierarchy levels for a list of GO-terms (y-axis) are shown in purple and the hierarchy levels for the maximal depth of paths in the GO-DAG passing through these GO-terms is shown in red. (**B**) Rank ordered GO-terms according to the score $s_t$.

For instance, in Fig. 5A, we show results for a list of enriched GO-terms of BP found from an analysis of the breast cancer gene regulatory network^27^. Specifically, the hierarchy levels (x-axis) of these GO-terms (y-axis) are shown in purple. For reasons of comparison, the maximal depth of paths in the GO-DAG passing through these GO-terms is shown in red. As one can see, in all cases, the GO-terms are not at the end of these paths but somewhere situated along the way toward the highest possible (maximal) level that can be reached by passing through the corresponding GO-terms. This information is important because on one-hand one wants to interrogate GO-terms that are biologically specific, i.e., are situated toward the highest hierarchy level of the GO-DAG - for human this would be level 19. On the other-hand not every GO-term is connected to the highest level, i.e., there is no path that would allow to reach the maximal level. Hence, there is a trade-off between absolute and relative position of a GO-term within a GO-DAG. For this reason, the GO-terms in Fig. 5A are ranked according to the distance between the two points (purple and red).

This trade-off can be formally quantified by the following score,

19 $$\begin{aligned} s_{t} = \text{ score } = \frac{ \text{ level }(GO)}{ \text{ level}_{max}(GO)} \times \frac{ \text{ level }(GO)}{ \text{ level}_{GO-DAG}(GO)} = p_1(\text{ max } \text{ path}) p_2(GO-DAG). \end{aligned}$$

Since the left-hand-side of Eq. (19), i.e, $\frac{ \text{ level }(GO)}{ \text{ level}_{max}(GO)} \in (0,1]$, as well as the right-hand-side, i.e., $\frac{ \text{ level }(GO)}{ \text{ level}_{GO-DAG}(GO)} \in (0,1]$ the resulting score is also positive and at most one. Hence, the score, $s_t$, is a product of two probabilities, i.e., $s_t = p_1(\text{ max } \text{ path}) p_2(GO-DAG)$ allowing to optimize the trade-off between both objectives.

The resulting score $s_t$ is shown in Fig. 5B. As one can see, the ranking of GO-terms is similar to Fig. 5A but not identical because Fig. 5A considers for the ranking only the relative distance between the actual and the maximal attainable position in a GO-DAG. Hence, both figures provide slightly complementary information. For our example GO:0006614 (SRP-dependent cotranslational protein targeting to membrane) and GO:0006613 (cotranslational protein targeting to membrane) have the highest score, which are interestingly directly connected in the GO-DAG. Overall, in general this information enables an exploratory analysis of GO-terms which complement the obtained p-values from an enrichment analysis.

In GOxploreR, such an analysis can be performed by using the commands *distRankingGO* and *scoreRankingGO*, i.e., the results in Fig. 5A,B can be obtained by

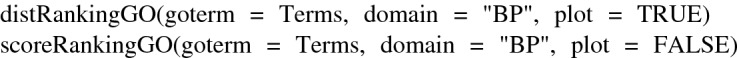



### Reduced GO-DAG

The starting point for many different types of analyses is usually a visualization of the data in order to derive an intuition about the information contained in the data. Unfortunately, for unfiltered GO-terms such a visualization is not feasible because the entire GO-DAG of an organism is too large containing thousands or even tens of thousands of GO-terms (see Table 1). For instance, even the smallest organism with respect to GO-terms of BP consists of 1491 nodes in the corresponding GO-DAG, distributed over 15 hierarchy levels. A graph of such a size cannot be visualized in an insightful way^28^. For this reason, we introduce a so called *reduced GO-DAG* that allows an easy visualization.

The underlying idea of such a reduced GO-DAG is a mapping from GO-terms into three node categories, namely: regular nodes (RN), jump nodes (JN) and leaf nodes (LN). A GO-term is called a ’regular node’ (RN) if all its children are on the next level, a GO-term is a ’jump node’ (JN) if it has children and at least one of these is not on the next level and a GO-term is a ’leaf node’ (LN) if it has no children at all. Such a mapping is obtain by the function *getGOcategory*.

**Figure 6:**
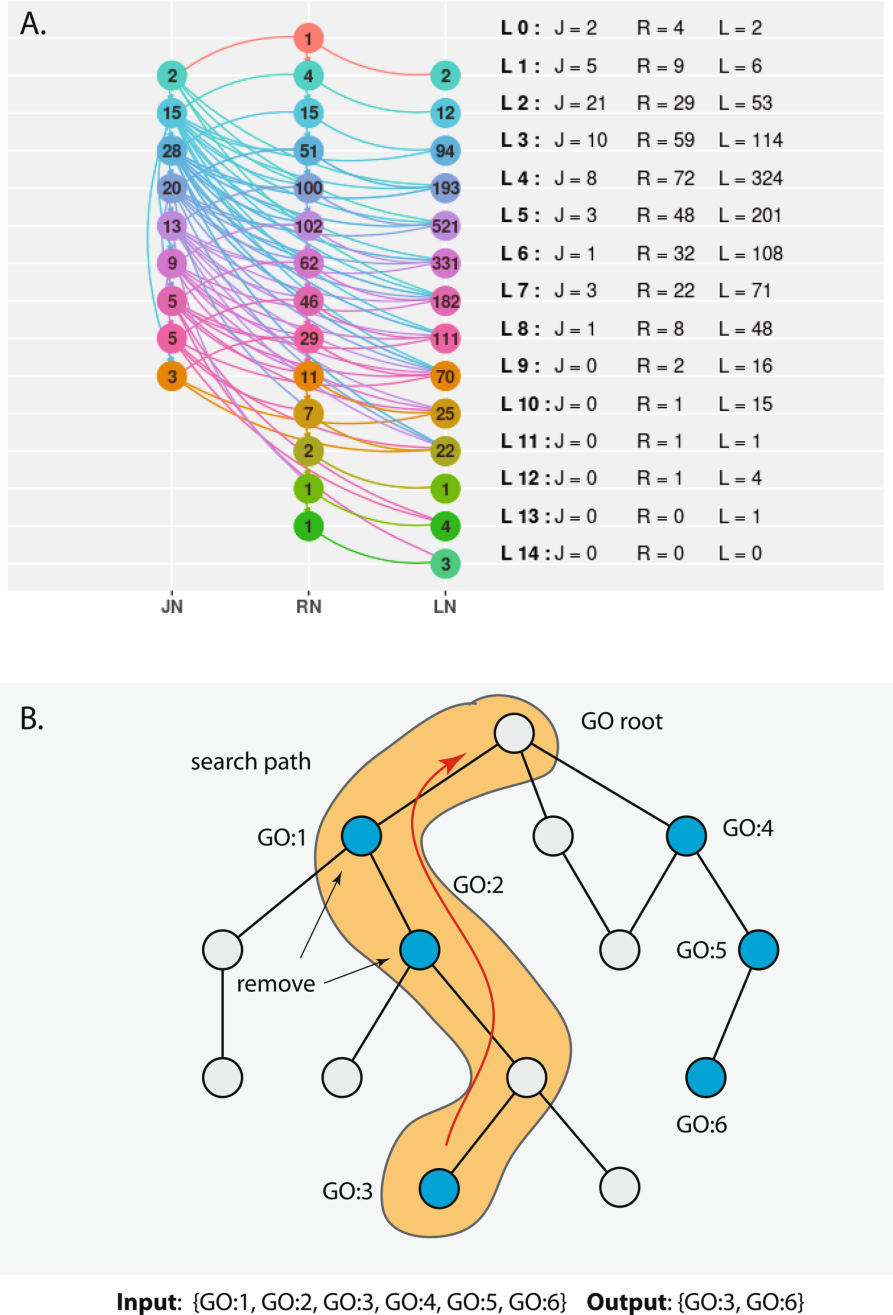
(**A**) Shown is a reduced GO-DAG of MF for *C. elegans.* The whole GO-DAG contains only 37 category nodes, i.e., RN, JN or LN and summarizes all 2103 GO-terms of MF for this organism. (**B**) Underlying idea for prioritizing GO-terms in a general DAG. Shown is one search path. Nodes in blue correspond to GO-terms in a given list.

As an example, Fig. 6A shows the reduced GO-DAG of MF for *C. elegans*. This GO-DAG contains only 37 category nodes, i.e., RNs, JNs or LNs, which summarize all 2102 GO-terms of MF for this organism on 14 hierarchy levels. That means only category nodes are shown that contain at least one GO-term, allowing a system-wide view of all MFs of *C. elegans*. Importantly, a reduced GO-DAG has the same number of hierarchy levels as the original GO-DAG because the mapping into category nodes does not effect the hierarchy levels. This holds for all GO-DAG. The following code demonstrates how the information shown in Fig. 6A can be obtained.

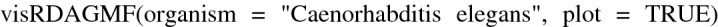



Similar visualizations are possible for all other organisms because even for human, there are only 52 (BP), 38 (MF), 43 (CC) nodes in the resulting reduced GO-DAG for the corresponding domains.

In case one has a list of GO-terms, one can also perform such a mapping only for this limited number of GO-terms. Furthermore, also a visualization for this sub-set of all GO-terms can be obtained using the function *visRDAGMF*. Overall, a reduced GO-DAG helps in simplifying the complexity provided by the gene ontology especially with respect to the connectivity between the GO-terms. This enables a general visualization for an exploratory analysis of system-wide information propagation capabilities.

### Prioritizing GO-terms

Finally, GOxploreR provides a function called *prioritizedGOTerms* for prioritizing GO-terms. The idea is to go beyond the ordering of GO-terms for a provided list of GO-terms to eliminate selected terms that are capturing redundant and less biologically specific information; see the discussion of Fig. 6B below.

In order to realize an implementation for such a function, we apply the following strategy (see Methods Sec. 2.4 for technical details). Specifically, it is known that the comparison of GO-terms with respect to their biological meaning is complex. However, the comparison of GO-terms that can be found along a path is much simpler because the higher a level of a GO-term, the more specific is its biological information^26^. That means traversing a path *vertically* toward higher levels increases the biological specificity of GO-terms implying that the GO-term at the end of a path is the most interesting one. Hence, by eliminating all GO-terms that are together on a path, except the one on the highest level, results in a prioritizing of terms with respect to the semantic meaning of GO-terms. The function *prioritizedGOTerms* implements this strategy. In Fig. 6B, we show visualized of this. Here one path is highlighted containing three GO-terms (GO:1, GO:2, and GO:3) whereas GO:3 has the highest level. This results in an elimination of GO:1 and GO:2. Similarly, all other paths are explored resulting in GO:1 and GO:6 as output of the prioritizing algorithm.

As an example, we investigate a list of GO-terms that was obtained from analyzing a gene regulatory network of *S. cerevisiae*^29^. The original list contains 30 different GO-terms of BP^29^, each significantly enriched with a significant p-value. Application of our function *prioritizedGOTerms* for prioritizing GO-terms results in only 5 GO-terms, shown in Table 2. Each of these 5 GO-terms is located on a separate brunch of the underlying GO-DAG between which no paths exist. Hence, despite of a certain similarity of the biological processes, e.g., for metabolic or mitochondrial processes, each of these terms is from a different, separate semantic category because otherwise connections with the DAG would exist. Such an analysis complements available p-values and gives further information on which GO-terms a follow-up analysis could focus on.

Overall, the function *prioritizedGOTerms* can prioritize a list of GO-terms with information about the semantic information content of a GO-DAG as provided by the level of GO-terms. If desired, a separate visualization could be obtained only for these GO-terms by using the function *visRDAGsubMF*.

**Table 2:** Using GOxploreR one can prioritize lists of GO-terms.

GO-term	GO-level	Description	*p* value	# genes
GO:0006364	9	rRNA processing	1.6e−39	237
GO:0032543	8	Mitochondrial translation	4.2e−167	100
GO:0044257	6	Cellular protein catabolic process	2.0e−78	347
GO:0019752	5	Carboxylic acid metabolic process	3.5e−67	370
GO:0007005	4	Mitochondrion organization	3.0e−168	282

The table shows results for significant GO-terms from analyzing a gene regulatory network of *S. cerevisiae*^29^ after the application of our prioritizing algorithm. The GO-terms are for BP and complement p-values obtained from an independent enrichment analysis.

## Discussion

In this paper, we introduced the R package GOxploreR and highlighted some of the functionality it provides. Overall, GOxploreR provides functions and algorithms for four different types of analyses. Specifically, GOxploreR enables a (1) direct access to structural features of GO, (2) structure-based ranking of GO-terms, (3) mapping to a reduced GO-DAG and (4) prioritizing of GO-terms.

The first three features of GOxploreR permit an exploratory analysis of GO-terms and GO-DAGs whereas the fourth feature provides a dedicated algorithm for a particular problem. Despite the fact that it is well-known that GO has the structure of a DAG, there are surprisingly few tools allowing a direct assess to structural, i.e., graph-based information of GO. Hence, our features and the corresponding functions help in utilizing this rich source of information which is in our opinion so far largely underexplored. A reason for this lack could be that the conceptual realization and implementation of graph-based algorithms is not straight forward requiring inter- and transdisciplinary knowledge of graphs and the underlying biology.

One important novelty of GOxploreR is to provide a mapping from a GO-DAG to a reduce GO-DAG. This leads to a tremendous reduction in complexity of graphs because a GO-DAG can contain thousands of nodes, depending on the organism and the domain, i.e., BP, MF or CC. In contrast, a reduced GO-DAG has at most three nodes of the categories, JN (jump node), RN (regular node) or LN (leaf node) on each hierarchy level. The idea behind this mapping is inspired by the detection of differentially expressed genes (DEG)^30^. While the expression level of a gene is continuous, a DEG analysis performs a kind of classification of the expression level into two categories: active and inactive. This allows a reduction in the complexity of the gene expression level by capturing simplified yet essential information. Our mapping from a GO-DAG to a reduce GO-DAG follows a similar strategy by capturing simplified yet essential information of the connection between GO-terms. As far as we know, GOxploreR is the only package that provides such a mapping and reduction in the GO complexity.

Another novelty of the GOxploreR package is to provide visualizations of reduced GO-DAGs. This feature is directly enabled by the tremendous reduction in complexity of the mapping from a GO-DAG to a reduce GO-DAG because the visualization of a DAG containing thousands of nodes (see Table 1) is not feasible. In contrast, a reduce GO-DAG permits such a visualization allowing to obtain an overview of the biological information processing of the entire ontology. Given the novelty of a mapping from a GO-DAG to a reduce GO-DAG other packages that provide also visualization capabilities do not offer this particular visualization.

Finally, the GOxploreR package provides a prioritizing algorithm. The idea of this algorithm is to go beyond the ordering of GO-terms for a given list of GO-terms, and to eliminate GO-terms capturing redundant biological information. For the prioritizing of GO-terms in a list, we utilized the fact that the higher a level of a GO-term is the more specific is its biological information^26^. That means *vertically* one wants to traverse a DAG as far down as possible because the end of a path is most specific compared to all other GO-terms along this path. Our algorithm applies this logic iteratively by starting from the GO-term at the highest level and searches all (shortest) paths to the root node. Then all GO-terms along these shortest paths are removed from the list and the procedure starts over; see Fig. 1 for a visualization. As a result, one obtains a prioritizing of GO-terms that is a parameter- and assumption-free algorithm which removes redundant GO-terms by exploiting only vertical structural information of a GO-DAG. Hence, the output of our prioritizing algorithm is a non-redundant ranking of GO-terms.

We would like to highlight that there is a crucial difference between our prioritizing algorithm and approaches based on the semantic similarity of genes^31,32^. The difference is that we utilize only vertical information from a GO-DAG. This implies that there is no need for comparing GO-terms horizontally because they cannot be connected by any path (besides over the root node). However, this horizontal comparison is usually the problem since the biological significance of different GO-terms on the same hierarchy level can be different. This simplifies the analysis yet allows the elimination of redundant GO-terms. The resulting list of GO-terms maybe be further reduced, however, not without making additional assumptions, e.g., in the form of semantic similarity measures. A common problem with the latter is that there is not one but many different measures for semantic similarity all of which are non-trivial in their definition and interpretation^33^. In contrast, our prioritizing algorithm is parameter- and assumption-free allowing to remove redundant GO-terms by exploiting only vertical structural information along paths of a GO-DAG. Another fundamental difference between our prioritizing algorithm and semantic similarity measures is that our algorithm focuses on GO-terms and not on genes. This facilitates a general systems view on the underlying problem from which the GO-terms have been obtained as represented by systems biology^34,35^.

In Table 3, we compare the capabilities of the GOxploreR package with other software tools available for analyzing GO. The first column shows the name of the software whereas the remaining columns refer to various features. Specifically, the second column indicates if a software tool is available as an R package and the third column refers to direct assess of structural information provided by a GO-DAG. Examples thereof are the hierarchical level of a GO-term, the GO-terms on a certain hierarchy level or the adjacency matrix of a DAG. The fourth column is about identifying the enrichment of GO terms, whereas the fifth column is about the availability of reduced GO-DAGs and the sixth column refers to a prioritizing algorithm for a list of GO-terms.

As one can see from Table 3, the GOxploreR package is considerably different from all the other software tools, hence, providing novel and complementary analyses functionality. Importantly, GOxploreR is available as R package allowing the easy utilization of it within existing analysis pipelines for their extensions. Hence, GOxploreR does not provide dead-end functionality via web-interfaces but enables future biomedical data science projects^36^.

**Table 3:** A comparison of the capabilities of various software tools for analyzing GO.

**Name**	R Package	Direct structural information to GO	Enrichment	Reduced GO-DAG	Prioritizing GO-terms
GOxploreR	Yes	Yes	No	Yes	Yes
OntologyTraverser^37^	Yes	Partly	Yes	No	No
Categorizer^38^	No	No	Yes	No	No
G-SESAME^39^	No	No	No	No	No
GOrilla^40^	No	No	Yes	No	No
GOGrapher^41^	No	Partly	No	No	No
agriGO^42^	No	No	Yes	No	No
topGO^43^	Yes	Yes	Yes	No	No
GOdb^44^	Yes	Yes	No	No	No

## Conclusion

In this paper, we introduced the R package GOxploreR, available from CRAN (after acceptance of the paper). GOxploreR is a versatile tool that can be applied to any list of GO-terms from an upstream analysis as a result from studying, e.g., differentially expressed genes, GWAS, biomarkers, gene sets or gene regulatory network studies^7–13^. Its main features include:

1. A direct access to structural features of GO.
2. A structure-based ranking of GO-terms.
3. A mapping from a GO-DAG to a reduced GO-DAG.
4. A visualization of reducuded GO-DAGs.
5. An algorithm for prioritizing GO-terms.

Given the lack of tools for exploring the DAG-structure of GO from a graph theoretical perspective, GOxploreR complements non-structural analysis tools. Overall, GOxploreR has the potential to enhance studies investigating differentially expressed genes, GWAS (genome-wide association study), biomarkers, gene sets or gene regulatory network studies significantly because the obtained information has a clear interpretation directly derived from the gene ontology knowledge base and is not based on additional assumptions.

## Supplementary information

Supplementary Information 1.

Supplementary Information 2.
