# Supplementary material for: Graph-based exploitation of gene ontology using GOxploreR for scrutinizing biological significance
Source: Sci Rep. 2020 Oct 7;10:16672. doi: 10.1038/s41598-020-73326-3 (PMC7542435; doi:10.1038/s41598-020-73326-3)
Supplement: Supplementary file 2 — Supplementary Information 2. [file 41598_2020_73326_MOESM2_ESM.pdf]

Supplementary file: Graph-based exploitation of Gene Ontology using GOxploreR for scrutinizing biological significance

Kalifa Manjang[1], Shailesh Tripathi[1], Olli Yli-Harja[2,3,6], Matthias Dehmer[4,5] and Frank Emmert-Streib[1,6*]

[1]Predictive Society and Data Analytics Lab, Faculty of Information Technology and Communication Sciences, Tampere University, Tampere, Finland
[2]Computational Systems Biology, Tampere University, Tampere, Korkeakoulunkatu 10, 33720, Tampere, Finland
[3]Institute for Systems Biology, Seattle, WA, United States
[4]Department of Biomedical Computer Science and Mechatronics, UMIT-The Health and Life Science University, 6060 Hall in Tyrol, Austria
[5]College of Artificial Intelligence, Nankai University, Tianjin 300350, China
[6]Institute of Biosciences and Medical Technology, 33520 Tampere, Finland

1 Determing the GO-DAG

The problem with existing packages is that none allows to obtain a GO-DAG for a domain, i.e., BP, MF or CC, directly in the form of an adjacency matrix. Instead, they provide local information which needs to be used to *deduce* such a tree. For instance, GOdb provides the function `GOBPCHILDREN` to get the children of a GO term for BP. For the other two domains similar functions are available. The problem is that a children node does not need to be on the next hierarchy level but can jump further down. For an example see Fig. 1.

The following example demonstrates how one can deduce a GO-DAG from this information. First, we list all children of a GO term (as obtained via the command `GOBPCHILDREN`).

$$CH(x_1) = \{x_2, x_3\} \tag{1}$$
$$CH(x_2) = \{x_8\} \tag{2}$$
$$CH(x_3) = \{x_4, x_5\} \tag{3}$$
$$CH(x_4) = \{x_6, x_7\} \tag{4}$$
$$CH(x_5) = \emptyset \tag{5}$$
$$CH(x_6) = \{x_8, x_9\} \tag{6}$$
$$CH(x_7) = \emptyset \tag{7}$$
$$CH(x_8) = \emptyset \tag{8}$$
$$CH(x_9) = \emptyset \tag{9}$$

The root node is unique and we assign it the level 0, i.e., $L(x_1) = 0$. The children for the root node receive as first assignment for a level the value $L(x_1) + 1 = 1$, i.e.,

$$L(x_2) = \{1\} \tag{10}$$
$$L(x_3) = \{1\} \tag{11}$$

We wrote the right-hand side as a set because if such a node appear again, we just add the new level value

to this set. Going through the list of children, we assign each children of a node x_i the value $L(x_i) + 1$.

$$CH(x_2) \rightarrow L(x_8) = \{2\} \tag{12}$$
$$CH(x_3) \rightarrow L(x_4) = \{2\},\, L(x_5) = \{2\} \tag{13}$$
$$CH(x_4) \rightarrow L(x_6) = \{3\},\, L(x_7) = \{3\} \tag{14}$$
$$CH(x_6) \rightarrow L(x_8) = \{2, 4\},\, L(x_9) = \{4\} \tag{15}$$
$$\tag{16}$$

From the last line we see that x_8 appears once on level 2 and once on level 4, which is correct if one looks at Fig. 1. However, there is just one correct level for x_8 and this is level 4. In general, if more than one level is assigned to a node then the correct one is the maximal of these values.

Such a GO-DAG can be constructed for every domain, i.e., biological process, molecular function and cellular component. In our package, we call the resulting graphs:

- `g.GO-DAG.BP`: A DAG for all GO-terms of biological processes
- `g.GO-DAG.MF`: A DAG for all GO-terms of molecular functions
- `g.GO-DAG.CC`: A DAG for all GO-terms of cellular components

2 Organism-specific GO-DAG

Starting from a GO-DAG for a domain, as constructed in the previous section and using a list of all genes from an organisms, we can map these genes to GO-terms. For a particular organism, not all GO-terms may be present but only a subset. Such a subset can then be mapped back to the entire GO-DAG of the knowledge base. This gives a subtree of the general GO-DAG that is organism-specific. Using the function `GetDAG(organism = o.name, domain = "BP")` one obtains, e.g., a GO-DAG of BPs for the organism given by 'o.name'. For all domains, the functions

- `GetDAG(organism = o.name, domain = "BP")`: A sub-DAG for all GO-terms of biological processes for organism 'o.name'.
- `GetDAG(organism = o.name, domain = "MF")`: A sub-DAG for all GO-terms of molecular functions for organism 'o.name'.
- `GetDAG(organism = o.name, domain = "CC")`: A sub-DAG for all GO-terms of cellular components for organism 'o.name'.

3 Reduced GO-DAG

Visualizing one of the GO-DAGs determined above (for all GO-terms or for organism-specific GO-terms) is usually challenging because of the size of such graphs containing thousands of GO-terms corresponding to nodes in a graph. For this reason, we derive a simplified GO-DAG, containing only dozens of nodes, that can be easily visualized to obtain a global overview of all used GO-terms.

In order to simplify a GO-DAG, we introduce the following categorization of GO-terms, including the root node. This categorization is applied to each level separately:

- A GO-term is in category 'leaf node' (LN) if it has no children.
- A GO-term is in category 'regular node' (RN) if all its children are on the next level.

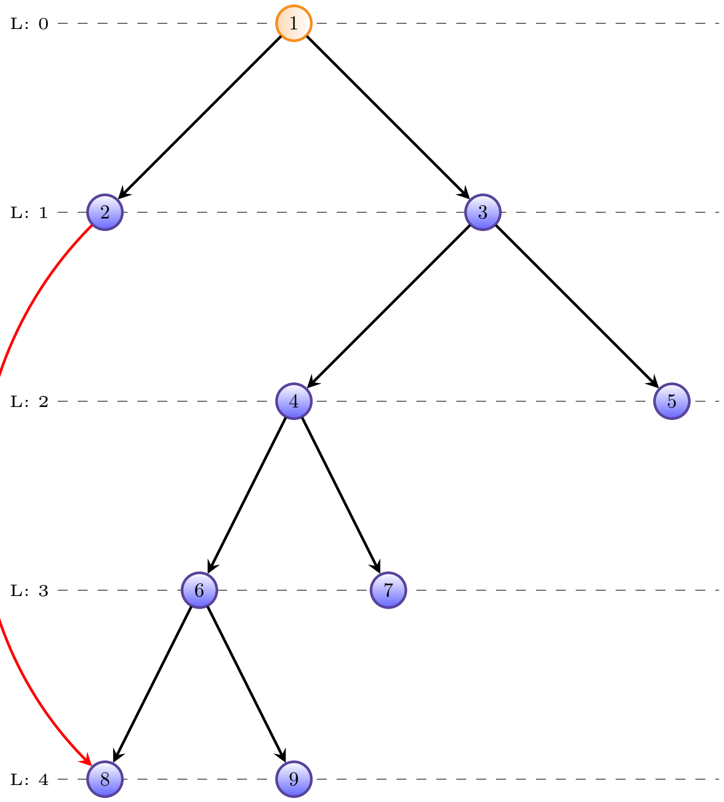

Figure 1. An example for a toy GO-DAG containing 9 GO-terms, whereas each node corresponds to one GO-term. The children of a node can jump over levels, as shown in red for the connection between node 2 and 8.

- A GO-term is in category 'jump node' (JN) if it has children and at least one of these is not on the next level.

We apply this categorization for all GO-terms. This results in the mapping

GO-term $X \to$ GO-term category on level L

That means we have functions of the form

$$(c, l) = f(X) \tag{17}$$

with $c \in \{\text{LN, RN, JN}\}$ and $l \in \mathbb{N}$. For instance, from Fig. 1 follows 3 $\to$ RN on level 1 and 2 $\to$ JN on level 1, which can be written formally as

$$(\mathrm{RN}, 1) = f(3) \tag{18}$$
$$(\mathrm{JN}, 1) = f(2) \tag{19}$$

Algorithmically, this is described in 1.

In addition to the node categorization, we need to find the connections between these nodes. This is obtained via Algorithm 2.

Algorithm 1: Categorization of GO-terms

For a GO-DAG with L levels, M nodes, adjacency matrix $A \in \mathbb{R}^{M \times M}$ and level function $l = g(i)$ for $i \in \{1, \ldots, M\}$ and $l \in \{0, \ldots, L\}$
Initialize hash H # for nodes in GO-DAG
Initialize hash V # for nodes in simplified GO-DAG
Initialize hash F
Initialize matrix $C \in \mathbb{R}^{(L+1) \times 3}$
Initialize vectors Ca, ca, h
for $i \in \{1, \ldots, M\}$ **do**
    $S = links(A(i,))$ # find all nodes S linking from i (outgoing links from i)
    $l_i = g(i)$
    $K = \emptyset$
    foreach *node* $j \in S$ **do**
        $l_j = g(j)$ # find the level of node j
        $K \leftarrow l_j$
    if $S = \emptyset$ **then**
        c_i =LN
    else if l_j *exists in* K *with* $l_j > l_i + 1$ **then**
        c_i =JN
    else if $|S| > 0$ **then**
        c_i =RN
    set $H\{(c_i, l_i)\} \leftarrow i$ # store set of nodes i with c_i and l_i
    set $Ca(i) = c_i$ # categorize node i
$k = 1$ # node ID for nodes in simplified GO-DAG
for $l \in \{0, \ldots, L\}$ **do**
    # summarize nodes of the same category
    $C(l, 1) = |H\{(\text{LN}, l)\}|$ # number of leaf nodes on level l
    $C(l, 2) = |H\{(\text{RN}, l)\}|$
    $C(l, 3) = |H\{(\text{JN}, l)\}|$
    foreach $C(l, c) > 0$ **do**
        set $V\{k\} = H\{(c, l)\}$ # mapping between old and new node IDs
        set $F\{(c, l)\} = k$
        set $h(k) = l$ # level function of simplified GO-DAG
        set $ca(k) = c$
        $k = k + 1$
$N = |V|$ # number of nodes in simplified GO-DAG

Algorithm 2: CALCULATE NUMBER OF LINKS BETWEEN CATEGORY NODES.

For A, F, M, N, h and Ca; see Algo 1
Initialize adjacency matrix B with $B \in \mathbb{R}^{N \times N}$ for simplified GO-DAG
for $l_1 \in \{0, \ldots, L\}$ **do**
    foreach *node* x *on level* l_1 **do**
        $c_1 = Ca(x)$ # find the category of node x
        $i_1 = F\{(c_1, l_1)\}$
        $S = Ch(x)$ # find all children of x using A
        foreach $y \in S$ **do**
            $c_2 = Ca(y)$ # find the category of node y
            $l_2 = g(y)$ # find the level of node y
            $i_2 = F\{(c_2, l_2)\}$
            $B(i_1, i_2) = B(i_1, i_2) + 1$

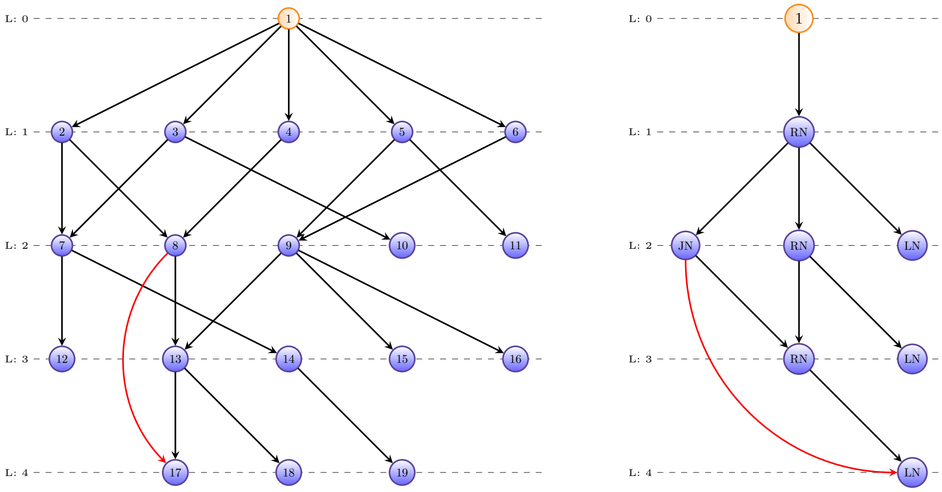

Figure 2. An example for the construction of a reduced GO-DAG. Left: An ordinary GO-DAG with 19 GO terms is shown. Right: The reduced GO-DAG with 8 nodes summarizes the left graph. Note, the nodes in the left graph are no GO-terms but node categories, i.e., either RN, JN or LN.

Overall, a GO-DAG is described by adjacency matrix A and level function g and analogously, a reduced GO-DAG is described by adjacency matrix B and level function h and C (number of original nodes summarized by a new category).

In Fig. 2 we show a complete example for this transformation. The GO-DAG on the left-hand side has 19 GO terms and the resulting simplified GO-DAG on the right-hand side has only 8 nodes, whereas these nodes corresponds to the three GO categories (RN, JN & LN) defined above. As one can see, each level will contain at most 3 nodes because this is the number of different categories. However, it is possible to have even fewer nodes, if a category is absent.

Importantly, this transformation can be applied to any GO-DAG regardless if it is for all GO terms of, e.g., BPs, or for a species-specific GO-DAG.

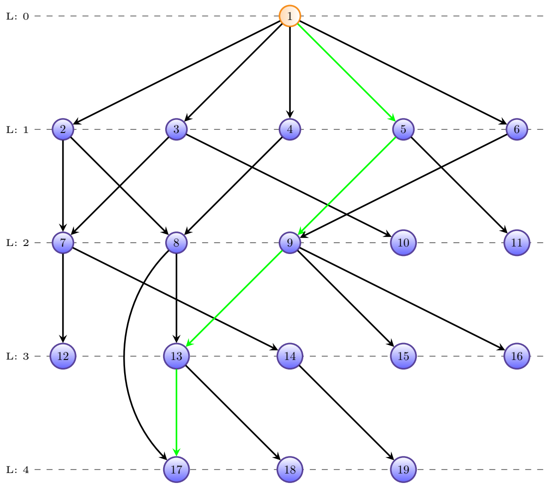

Figure 3. Shown is a path (green) in a GO-DAG, where nodes correspond to GO-terms. Along this path, the biological semantics increases from node to node the further down one traverses the path.

4 Prioritizing lists of GO-terms

Overall, the comparison of GO-terms with respect to their biological semantic importance is complex. However, the comparison of GO-terms along a path is simple because the higher a level of a GO-term is the more specific is its biological information [1]. That means *vertically* one wants to traverse a DAG along a path as far down as possible. This implies that the GO-term at the end of a path is most interesting compared to all other GO-terms along this path. This increase in the semantic meaning along *vertical* paths is exploited by our algorithm for prioritizing lists of GO-terms.

Our algorithm applies the above described logic iteratively, by starting from the GO-term at the highest level and searches all shortest paths to the root node. Then all GO-terms along these shortest paths are removed from the list and the procedure starts over. As a result, one obtains a parameter-free, non-redundant ranking of GO-terms that exploits only vertical structural information of GO. The pseudo-code of this is shown in Algorithm 3. Here XX corresponds to BP, MF or CC. The algorithm guarantees that for a non-empty list, H, of GO-terms the resulting set, R, containing the prioritized GO-terms consists of at least one GO-term.

References

1. G. Dennis, B. T. Sherman, D. A. Hosack, J. Yang, W. Gao, H. C. Lane, and R. A. Lempicki. DAVID: Database for Annotation, Visualization, and Integrated Discovery. *Genome Biology*, 4(5):R60, 2003.

Algorithm 3: PRIORITIZING A LIST OF GO-TERMS.

For a list, H, of GO-terms in domain XX, a GO-DAG of XX and level function g
Initialize a list R
$n = |H|$
foreach $i \in H$ **do**
  $l_i = g(i)$ # find level for each GO-term
while $n > 0$ **do**
  r =rank($\{l_i|H\}$) # ranking of all $\{l_i\}$ that are in H from high to low
  $R \leftarrow \arg(r_1)$ # GO-term that belongs to the highest rank
  for $\arg(r_1)$ find shortest path(s), p, to root
  delete all nodes in H that are on $p\backslash \arg(r_1)$
  $n = |H|$
R contains the prioritized GO-terms.

Introduction to the GOxploreR package

Kalifa Manjang, Shailesh Tripathi, Olli Yli-Harja, Matthias Dehmer & Frank Emmert-Streib

31 July 2020

Contents

Introduction . . . 3
Overview of the functionality of the package . . . 3
Gene2GOTermAndLevel . . . 3
Gene2GOTermAndLevel_ON . . . 5
GOTermXXOnLevel . . . 5
Level2GOTermXX . . . 6
Level2LeafNodeXX . . . 6
Level2JumpNodeXX . . . 7
Level2RegularNodeXX . . . 7
Level2NoLeafNodeXX . . . 7
getGOcategory . . . 8
degreeDistXX . . . 8
GOTermXX2ChildLevel . . . 10
GetLeafNodesXX . . . 10
GO2DecXX . . . 11
GetDAG . . . 11
visRDAGXX . . . 12
visRsubDAGXX . . . 14
distRankingGO . . . 15
scoreRankingGO . . . 17
prioritizedGOTerms . . . 18
GO4Organism . . . 19
Conclusion . . . 20
References . . . 20

List of Figures

1 Degree distribution of the biological process GO-terms on level 4. . . . 9
2 Degree distribution of the molecular function GO-terms on level 2. . . . 9
3 Degree distribution of the cellular component GO-terms on level 10. . . . 10
4 Visualization of a reduced GO-DAG for Caenorhabditis elegans. . . . 14
5 Visualization of a reduced sub-GO-DAG of BPs for Human . . . 15
6 The hierarchy levels for a list of GO-terms (y-axis) are shown in purple and the hierarchy levels for the maximal depth of paths in the GO-DAG passing through these GO-terms is shown in red. 17

List of Tables

1 Organisms supported by the GOxploreR package. . . . 3

Introduction

The GOxploreR package is an R package that provides a simple and efficient way to communicate with the gene ontology (GO) database. The gene ontology is a major bioinformatics initiative by the gene ontology consortium. The goal is to categorize the gene and gene product function. The ontology is structured into three distinct aspects of gene function: molecular function (MF), cellular component (CC), and biological process (BP) together with over 45,000 terms and 130,000 relations whereas the majority of information is centered around ten model organisms [1]. In addition, GO includes annotations by linking specific gene products to GO-terms. Currently, GO is the most comprehensive and widely used knowledgebase concerning functional information about genes.

Organism	Name used as options in the package	Number of genes
Human	"Homo sapiens" / "Human"	19155
Mouse	"Mus musculus" / "Mouse"	20929
Caenorhabditis elegans	"Caenorhabditis elegans" / "Worm"	14697
Drosophila melanogaster	"Drosophila melanogaster" / "Fruit fly"	12683
Rat	"Rattus norvegicus" / "Rat"	19383
Baker's yeast	"Saccharomyces cerevisiae" / "Yeast"	5502
Zebrafish	"Danio rerio" / "Zebrafish"	20718
Arabidopsis thaliana	"Arabidopsis thaliana" / "Cress"	25891
Schizosaccharomyces pombe	"Schizosaccharomyces pombe" / "Fission yeast"	5055
Escherichia coli	"Escherichia coli" / "E.coli"	3449

Table 1: Organisms supported by the GOxploreR package.

This vignette gives an overview of the functionality provided by the GOxploreR package.

The package is freely available on CRAN and can be installed using the following command:

```
install.packages("GOxploreR")
```

The package function can be loaded using:

```
library(GOxploreR)
```

Note that the package needs to be installed to be loaded.

Overview of the functionality of the package

The following is a brief description of the package functionality.

Gene2GOTermAndLevel

The Gene2GOTermAndLevel function provides information associated with a list of genes. Given a gene or a list of genes, an organism, and a domain (BP, MF or CC) the function provides the Gene Ontology terms (GO-terms) associated with the genes and their respective levels of the DAG. The default argument of the domain is BP. For the arguments of the option 'organism' see Table 1.

```
# The cellular component gene ontology terms will be retrieve and their levels
Gene2GOTermAndLevel(genes = c(10212, 9833, 6713), organism = "Homo sapiens", domain = "CC")
```

```
#>    Entrezgene ID      GO ID Domain Level
#> 1          10212 GO:0005634     CC     7
#> 2          10212 GO:0005737     CC     5
#> 3          10212 GO:0016607     CC    13
#> 4          10212 GO:0016020     CC     1
#> 5          10212 GO:0005654     CC    10
#> 6          10212 GO:0000346     CC     9
```

```
#> 7           9833 GO:0016020     CC     1
#> 8           9833 GO:0005886     CC     4
#> 9           9833 GO:0005634     CC     7
#> 10          9833 GO:0005737     CC     5
#> 11          9833 GO:0005938     CC     8
#> 12          6713 GO:0016020     CC     1
#> 13          6713 GO:0016021     CC     4
#> 14          6713 GO:0005783     CC     7
#> 15          6713 GO:0005789     CC    10
#> 16          6713 GO:0031090     CC     3
#> 17          6713 GO:0043231     CC     6
```

```r
# The biological process gene ontology terms will be retrieve and their levels
Gene2GOTermAndLevel(genes = c(100000642, 30592, 58153, 794484), organism = "Danio rerio")
```

```
#>    Entrezgene ID      GO ID Domain Level
#> 1      100000642 GO:0007186     BP     5
#> 2          30592 GO:0045214     BP     8
#> 3          30592 GO:0060047     BP     5
#> 4          30592 GO:0060038     BP     9
#> 5          30592 GO:0048738     BP     7
#> 6          30592 GO:0055005     BP    11
#> 7          30592 GO:0055015     BP    10
#> 8          30592 GO:0055004     BP    11
#> 9          30592 GO:0045823     BP     7
#> 10         58153 GO:0034755     BP     9
#> 11         58153 GO:0006811     BP     4
#> 12        794484 GO:0008150     BP     0
```

```r
# The molecular function gene ontology terms will be retrieve and their levels
Gene2GOTermAndLevel(genes = c(100009600, 18131, 100017), organism = "Mouse", domain = "MF")
```

```
#>    Entrezgene ID      GO ID Domain Level
#> 1      100009600 GO:0008270     MF     6
#> 2      100009600 GO:0043565     MF     5
#> 3      100009600 GO:0046872     MF     4
#> 4      100009600 GO:0003677     MF     4
#> 5          18131 GO:0005515     MF     2
#> 6          18131 GO:0005509     MF     5
#> 7          18131 GO:0038023     MF     2
#> 8          18131 GO:0042802     MF     3
#> 9          18131 GO:0019899     MF     3
#> 10        100017 GO:0005515     MF     2
#> 11        100017 GO:0035650     MF     3
#> 12        100017 GO:0001784     MF     5
#> 13        100017 GO:0005102     MF     3
#> 14        100017 GO:0005546     MF     8
#> 15        100017 GO:0001540     MF     4
#> 16        100017 GO:0050750     MF     5
#> 17        100017 GO:0030159     MF     4
#> 18        100017 GO:0030276     MF     3
#> 19        100017 GO:0035612     MF     3
#> 20        100017 GO:0035591     MF     3
#> 21        100017 GO:0035615     MF     5
```

Gene2GOTermAndLevel_ON

This function is similar to the Gene2GOTermAndLevel function, the only difference is that this function queries the Ensembl database online (ON) for GO-terms (making it relatively slow). That means the results from the Gene2GOTermAndLevel_ON function are always up to date but an internet connection is needed for its execution. This function does not provide support for Escherichia coli.

```
# The cellular component gene ontology terms will be retrieve and their levels
Gene2GOTermAndLevel_ON(genes = c(10212, 9833, 6713), organism = "Homo sapiens", domain ="CC")

# The biological process gene ontology terms will be retrieve and their levels
Gene2GOTermAndLevel_ON(genes = c(100000711, 100000710, 100000277), organism = "Danio rerio")

# The molecular function gene ontology terms will be retrieve and their levels
Gene2GOTermAndLevel_ON(genes = c(100009609, 100017, 100034361), organism = "Mouse", domain = "MF")
```

GOTermXXOnLevel

This function gives the level of a GO-term based on a DAG. The results for organism-specific GO-DAGs are the same as for the general GO-DAG. The XX in the name above should be replaced by either BP, MF, or CC.

```
# Retrieve the level of a GO biological process term
goterms <- c("GO:0009083","GO:0006631","GO:0006629","GO:0014811","GO:0021961")
GOTermBPOnLevel(goterm = goterms)
```

```
#>          Term Level
#> 1 GO:0009083     8
#> 2 GO:0006631     7
#> 3 GO:0006629     3
#> 4 GO:0014811    19
#> 5 GO:0021961    15
```

```
# Retrieve the level of a GO molecular function term
goterms <- c("GO:0005515","GO:0016835","GO:0046976","GO:0015425","GO:0005261")
GOTermMFOnLevel(goterm = goterms)
```

```
#>          Term Level
#> 1 GO:0005515     2
#> 2 GO:0016835     3
#> 3 GO:0046976     9
#> 4 GO:0015425    14
#> 5 GO:0005261     7
```

```
# Retrieve the level of a GO cellular component term
goterms <- c("GO:0055044","GO:0030427","GO:0036436","GO:0034980","GO:0048226")
GOTermCCOnLevel(goterm = goterms)
```

```
#>          Term Level
#> 1 GO:0055044     1
#> 2 GO:0030427     3
#> 3 GO:0036436    16
#> 4 GO:0034980     9
#> 5 GO:0048226     8
```

Level2GOTermXX

This function gives all the GO-terms from a given GO-level. These GO-terms can be from the general GO-DAG or from an organism-specific GO-DAG. If the "organism" argument is given, the GO-terms will be acquired from the organism's (organism supported by the package) DAG level, However, if no value for the "organism" parameter is given then the general GO-DAG is used (default). The XX in the name should be replaced by either BP, MF, or CC.

```r
# Retrieve all the GO-terms from a particular GO BP level
Level2GOTermBP(level = 1, organism = "Human")
```

```
#>  [1] "GO:0002376" "GO:0048511" "GO:0008283" "GO:0023052" "GO:0032502"
#>  [6] "GO:0009987" "GO:0008152" "GO:0007610" "GO:0040011" "GO:0098743"
#> [11] "GO:0000003" "GO:0001906" "GO:0043473" "GO:0050896"
```

```r
# Retrieve all the GO-terms from a particular GO MF level
Level2GOTermMF(level = 14, organism = "Rat")
```

```
#> [1] "GO:0005391" "GO:0008553" "GO:0086039" "GO:0046961" "GO:1905056"
#> [6] "GO:1905059" "GO:0008900"
```

```r
# Retrieve all the GO-terms from the general GO CC level
Level2GOTermCC(level = 16)
```

```
#> [1] "GO:0032777" "GO:0030085" "GO:0036436" "GO:0036437"
```

Level2LeafNodeXX

This function gives all the leaf nodes from a particular GO-level. Leaf nodes can also be attained from the organism-specific GO-DAG. The "organism" parameter is optional. If supplied, the leaf node from the respective organism's level will be acquired. The default is the general GO-DAG. The XX should be substituted with either BP, MF, or CC.

```r
# Get all leaf nodes from a GO BP level
Level2LeafNodeBP(level = 2, organism = "Danio rerio")
```

```
#>  [1] "GO:0032259" "GO:0030431" "GO:0032504" "GO:0035988" "GO:0060242"
#>  [6] "GO:1990845" "GO:0006807" "GO:0035726" "GO:0036268" "GO:0061744"
#> [11] "GO:0033058" "GO:0019835" "GO:0007624"
```

```r
# Get all leaf nodes from a GO MF level
Level2LeafNodeMF(level = 13)
```

```
#>  [1] "GO:0032778" "GO:0015421" "GO:0015423" "GO:0015410" "GO:0015413"
#>  [6] "GO:0015444" "GO:0015445" "GO:0015446" "GO:0015433" "GO:0015408"
#> [11] "GO:0044604" "GO:0008551" "GO:0008554" "GO:0015610" "GO:0015611"
#> [16] "GO:0015612" "GO:0015614" "GO:0015609" "GO:0033154" "GO:0033297"
#> [21] "GO:0031459" "GO:0046962" "GO:0046932" "GO:0046933" "GO:0016463"
#> [26] "GO:0102014" "GO:0102017"
```

```r
# Get all leaf nodes from a GO CC level
Level2LeafNodeCC(level = 15, organism = "Schizosaccharomyces pombe")
```

```
#>  [1] "GO:0031011" "GO:0000812" "GO:0036410" "GO:0042729" "GO:0005754"
#>  [6] "GO:0005745" "GO:0005756" "GO:0061617" "GO:1990467" "GO:1990468"
#> [11] "GO:0000274"
```

Level2JumpNodeXX

This function gives for a GO-level the GO-terms which correspond to jump Nodes (JNs). The JNs are GO-terms which have at least one child term not present in the level below the parent term. If no organism is given, the default is the general GO-DAG. The XX in the name should be substituted with BP, MF, or CC.

```
# All jump nodes from the GO BP level
head(Level2JumpNodeBP(level = 2, organism = "Homo sapiens"))
```

```
#> [1] "GO:0048066" "GO:0006955" "GO:0019835" "GO:0007568" "GO:0007623"
#> [6] "GO:0051301"
```

```
# All jump nodes from the GO MF level
head(Level2JumpNodeMF(level = 3, organism = "Homo sapiens"))
```

```
#> [1] "GO:0008134" "GO:0005102" "GO:0005548" "GO:0017081" "GO:0008047"
#> [6] "GO:0030276"
```

```
# All jump nodes from the GO CC level
head(Level2JumpNodeCC(level = 7, organism = "Homo sapiens"))
```

```
#> [1] "GO:0005887" "GO:0005856" "GO:0000307" "GO:0048471" "GO:0031410"
#> [6] "GO:0030425"
```

Level2RegularNodeXX

This function gives for a GO-level the GO-terms which correspond to regular Nodes (RNs). The RNs are those GO-terms whose child terms are all present in the level right below the parent's level. The XX in the name should be subsitituted with BP, MF, or CC.

```
# All regular nodes from the BP level
head(Level2RegularNodeBP(level = 9, organism = "Zebrafish"))
```

```
#> [1] "GO:0070588" "GO:0002088" "GO:0003128" "GO:0060956" "GO:0048593"
#> [6] "GO:0031076"
```

```
# All regular nodes from the MF level
head(Level2RegularNodeMF(level = 7, organism = "Homo sapiens"))
```

```
#> [1] "GO:0016887" "GO:0017048" "GO:0004708" "GO:0004950" "GO:0005088"
#> [6] "GO:0004939"
```

```
# All jump nodes from the CC level
head(Level2RegularNodeCC(level = 7))
```

```
#> [1] "GO:0031230" "GO:0031232" "GO:0031233" "GO:0031234" "GO:0031235"
#> [6] "GO:0031201"
```

Level2NoLeafNodeXX

This function gives all the GO-terms from a GO-level that are not leaf nodes. Similarly, all non-leaf GO-terms from an organism-specific DAG can be returned by providing the organism of interest. The default is the general GO-DAG. The XX in the name should be substituted with either BP, MF or CC.

```
# All GO-terms on a particular GO BP level that are not leaf nodes
Level2NoLeafNodeBP(level = 16, organism = "Homo sapiens")
```

```
#>  [1] "GO:0072540" "GO:0014808" "GO:0060314" "GO:0051281" "GO:0051280"
#>  [6] "GO:0045625" "GO:0045624" "GO:0031585" "GO:0021966" "GO:0045623"
```

```r
# All GO-terms on a particular GO MF level that are not leaf nodes
Level2NoLeafNodeMF(level = 11, organism = "Caenorhabditis elegans")
```

```
#> [1] "GO:0017116" "GO:0042625"
```

```r
# All GO-terms on a particular GO CC level that are not leaf nodes
Level2NoLeafNodeCC(level = 12, organism = "Homo sapiens")
```

```
#>  [1] "GO:0000784" "GO:0000118" "GO:0016591" "GO:0016604" "GO:0000790"
#>  [6] "GO:0030672" "GO:0000123" "GO:0030669" "GO:0030176" "GO:0000777"
#> [11] "GO:0035097" "GO:0043596" "GO:0000780" "GO:0008023" "GO:0032580"
#> [16] "GO:0000235" "GO:0000137" "GO:0005746" "GO:0000836" "GO:0017059"
#> [21] "GO:0005849" "GO:0012510" "GO:0031261" "GO:0098799" "GO:0005879"
#> [26] "GO:0030125" "GO:0005858" "GO:0031205" "GO:0098999" "GO:0000120"
#> [31] "GO:0005713" "GO:1990716" "GO:0001534" "GO:0097545"
```

getGOcategory

Given a GO-term or a list of GO-terms, this function returns the category of the term. The categories are jump nodes (JN), regular nodes (RN) and leaf nodes (LN).

```r
goterm <- c("GO:0009083","GO:0006631","GO:0006629","GO:0016835","GO:0046976","GO:0048226")

# Returns the categories of the GO-terms in the list
getGOcategory(goterm = goterm)
```

```
#>         Term Category Domain
#> 1 GO:0009083       JN     BP
#> 2 GO:0006631       JN     BP
#> 3 GO:0006629       JN     BP
#> 4 GO:0016835       RN     MF
#> 5 GO:0046976       LN     MF
#> 6 GO:0048226       LN     CC
```

degreeDistXX

This function obtains the degree distribution of the GO-terms on a GO-level. A bar plot is obtained which shows how many nodes in the GO-level have a certain degree k. The XX in the name should be substituted with either BP, MF, or CC.

```r
# Degree distribution of the GO-terms on a particular GO BP level
degreeDistBP(level = 4)
```

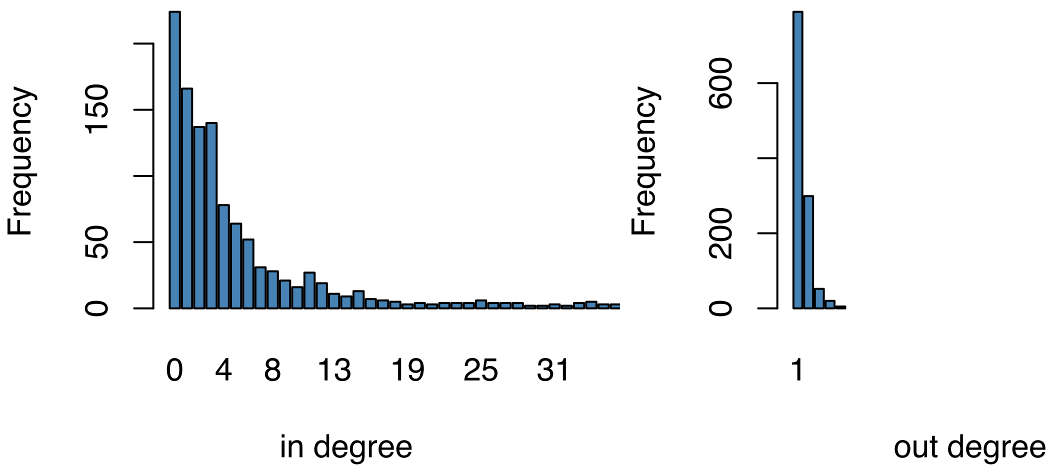

Figure 1: Degree distribution of the biological process GO-terms on level 4.

```
# Degree distribution of the GO-terms on a particular GO MF level
degreeDistMF(level = 2)
```

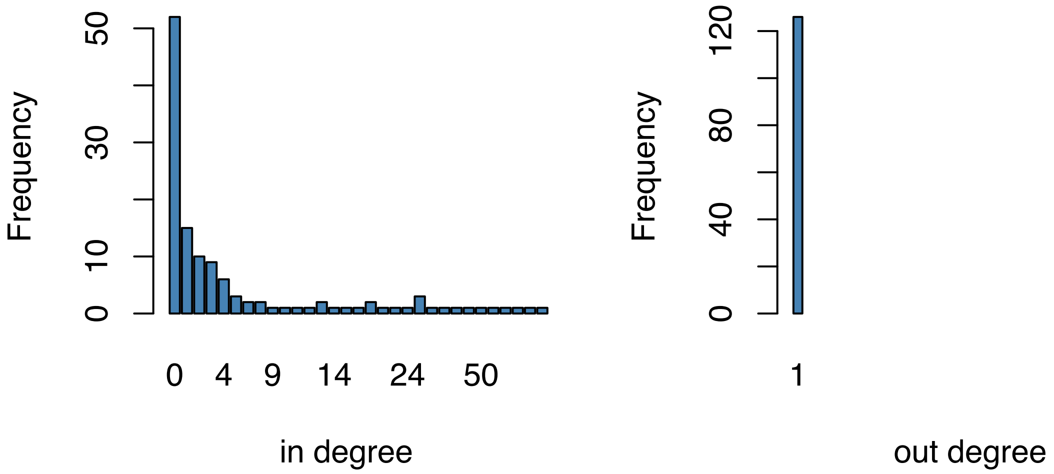

Figure 2: Degree distribution of the molecular function GO-terms on level 2.

```
# Degree distribution of the GO-terms on a particular GO CC level
degreeDistCC(level = 10)
```

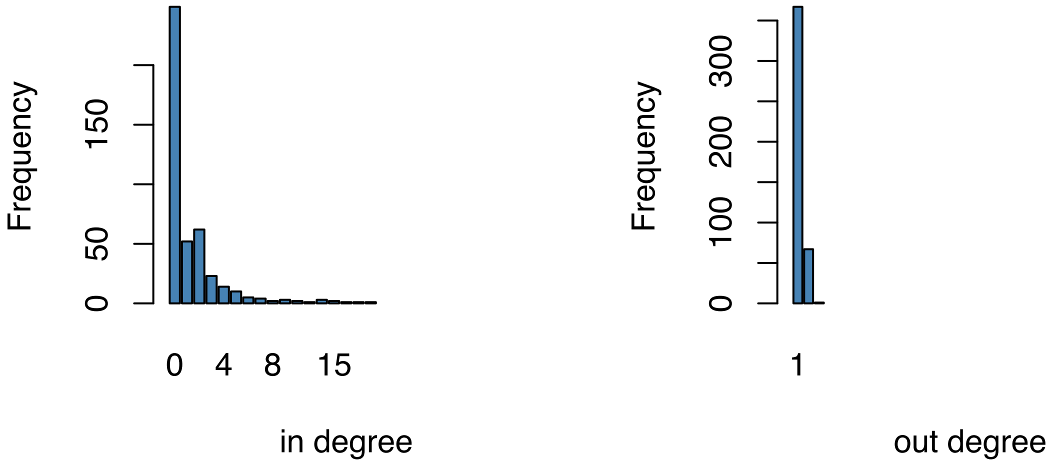

Figure 3: Degree distribution of the cellular component GO-terms on level 10.

GOTermXX2ChildLevel

For a GO-term it's children level are derived. The XX in the name should be substituted with BP, MF, or CC.

```
# Get the level of a GO BP term's children
GOTermBP2ChildLevel(goterm = "GO:0007635")
```

```
#> $Terms
#> [1] "GO:0007636" "GO:0007637" "GO:0042048" "GO:0061366"
#>
#> $Level
#> [1] 5 7 4 6
```

```
# Get the level of a GO MF term's children
GOTermMF2ChildLevel(goterm = "GO:0098632")
```

```
#> $Terms
#> [1] "GO:0086080" "GO:0098641"
#>
#> $Level
#> [1] 4 5
```

```
# Get the level of a GO CC term's children
GOTermCC2ChildLevel(goterm = "GO:0071735")
```

```
#> $Terms
#> [1] "GO:0071736" "GO:0071737"
#>
#> $Level
#> [1] 5 9
```

GetLeafNodesXX

This function gives all the leaf nodes of a certain organism. If the input value is empty or is "BP", "MF"" or "CC"" the default DAG is the general GO-DAG. The value for XX should be subsituted with BP, MF or CC.

```
# All leaf nodes from the GO BP tree
head(GetLeafNodesBP("BP"))
```

```
#>          Term Level
#> 1 GO:0140084     4
#> 2 GO:0000734     3
#> 3 GO:0000738     7
#> 4 GO:0140076     5
#> 5 GO:0140077     5
#> 6 GO:0140021     7
```

```r
# All leaf nodes from the GO MF tree
head(GetLeafNodesMF(organism = "Mouse"))
```

```
#>          Term Level
#> 1 GO:0031386     1
#> 2 GO:0090729     1
#> 3 GO:0015643     2
#> 4 GO:0046848     2
#> 5 GO:0005044     2
#> 6 GO:0008307     2
```

```r
# All leaf nodes from the GO CC tree
head(GetLeafNodesCC(organism = "Caenorhabditis elegans"))
```

```
#>          Term Level
#> 1 GO:0031974     1
#> 2 GO:0005623     1
#> 3 GO:0031594     2
#> 4 GO:0098981     2
#> 5 GO:0098982     2
#> 6 GO:0060076     2
```

GO2DecXX

This function returns all descedant child nodes of a GO-term. That means, we begin from a GO-term and find all the GO-terms of its children and their children until we reach all the way down of the DAG. The XX in the name should be substituted with BP, MF or CC.

```r
# Biological process GO-term descendant terms
GO2DecBP(goterm = "GO:0044582")
```

```
#> [1] "GO:1900497" "GO:1900498" "GO:1900499"
```

```r
# Molecular function GO-term descendant terms
GO2DecMF(goterm = "GO:0036442")
```

```
#> [1] "GO:0008553" "GO:0046961"
```

```r
# Cellular component GO-term descendant terms
GO2DecCC(goterm = "GO:0031233")
```

```
#> [1] "GO:0031237" "GO:0031362" "GO:0071575" "GO:1990914"
```

GetDAG

This function gives the GO-terms in the Gene Ontology as an edgelist corresponding to a directed acyclic graph (DAG) for the GO-terms of a certain organism. This can also be obtained for the general GO-DAG (not organism-specific).

```r
# Represent all the BP gene association GO-terms for human as an edgelist
head(GetDAG(organism = "Human", domain = "BP"))
```

```
#>      [,1]         [,2]
#> [1,] "GO:0008150" "GO:0000003"
#> [2,] "GO:0008150" "GO:0008152"
#> [3,] "GO:0008150" "GO:0001906"
#> [4,] "GO:0008150" "GO:0002376"
#> [5,] "GO:0008150" "GO:0040007"
#> [6,] "GO:0008150" "GO:0007610"
```

```
# Represent all the MF gene association GO-terms for Mouse as an edgelist
head(GetDAG(organism = "Mouse", domain = "MF"))
```

```
#>      [,1]         [,2]
#> [1,] "GO:0003674" "GO:0140110"
#> [2,] "GO:0003674" "GO:0003824"
#> [3,] "GO:0003674" "GO:0038024"
#> [4,] "GO:0003674" "GO:0045735"
#> [5,] "GO:0003674" "GO:0005198"
#> [6,] "GO:0003674" "GO:0005215"
```

```
# Represent all the CC gene association GO-terms for Caenorhabditis elegans as an edgelist
head(GetDAG(organism = "Caenorhabditis elegans", domain = "CC"))
```

```
#>      [,1]         [,2]
#> [1,] "GO:0005575" "GO:0016020"
#> [2,] "GO:0005575" "GO:0005576"
#> [3,] "GO:0005575" "GO:0005623"
#> [4,] "GO:0005575" "GO:0009295"
#> [5,] "GO:0005575" "GO:0030054"
#> [6,] "GO:0005575" "GO:0031974"
```

visRDAGXX

The visualization of a GO-DAG is difficult primarily because of the size of the graphs containing thousands of GO-terms. For this reason, we invented a simple method that combines GO-terms with similar characteristics together. This includes a global summary of all GO-terms in the DAG. Every node in the reduced DAG comprises 1 or more GO-terms and these GO-terms can be accessed by using certain information (i.e. the level and what type of node category they represent for example "RN", "JN" or "LN"). This is what we call the reduced GO-DAG for an organism. Furthermore, the function returns a list which contains the GO-terms in each category and the plot of the reduced DAG. To retrieve just the GO-terms in each category, the "plot" argument can be set to "FALSE" . The XX in the name should be substituted with BP, MF or CC. The total number of GO-terms in each node is represented by the node label. For instance, in Figure 4 , Level 0 (i.e "L0 RN") has 1 GO-term present in the node category.

The label "J","R" and "L" on the right side of Figure 4 gives the number of connections between the regular node (RN) on the level and the nodes right below it (RN are nodes that have all their children nodes represented in the next level). For example, on L1, The label J = 5, R = 9 and L = 6 means that the RNs on the level have 5 of it's children nodes as Jump nodes (JN) on L2, 9 of it's descendant are Regular nodes (RN) and 6 of its children GO-terms on L2 are leaf nodes (LN).

```
# The GO-terms in each node category of the reduced Caenorhabditis elegans GO-DAG
head(visRDAGMF(organism = "Caenorhabditis elegans", plot = FALSE))
```

```
#> $`L5 RN`
#>   [1] "GO:0003727" "GO:0061630" "GO:0004672" "GO:0043565" "GO:0019901"
#>   [6] "GO:0008135" "GO:0008234" "GO:0004519" "GO:0008236" "GO:0003729"
#>  [11] "GO:0003725" "GO:0004000" "GO:0008017" "GO:0019903" "GO:0035257"
#>  [16] "GO:0016791" "GO:0004540" "GO:0051287" "GO:0005160" "GO:0016505"
```

```
#>  [21] "GO:0035091" "GO:0005092" "GO:0004527" "GO:0031490" "GO:0001223"
#>  [26] "GO:0008237" "GO:0004175" "GO:0008081" "GO:0004407" "GO:0046914"
#>  [31] "GO:0004104" "GO:0004952" "GO:0030295" "GO:0004022" "GO:0008376"
#>  [36] "GO:0050660" "GO:0003997" "GO:0004620" "GO:0000268" "GO:0070815"
#>  [41] "GO:0071855" "GO:0005179" "GO:0008528" "GO:0072542" "GO:0004536"
#>  [46] "GO:0036002" "GO:0008238" "GO:0035673" "GO:0016407" "GO:0016307"
#>  [51] "GO:0004576" "GO:0015101" "GO:0015165" "GO:0008417" "GO:0015020"
#>  [56] "GO:0050661" "GO:0004368" "GO:0008375" "GO:0008378" "GO:0033744"
#>  [61] "GO:0000030" "GO:0004619" "GO:0004611" "GO:0017076" "GO:0016597"
#>  [66] "GO:0002094" "GO:0070569" "GO:0019843" "GO:0015929" "GO:0034979"
#>  [71] "GO:0000339" "GO:0008173" "GO:0051377" "GO:0004645" "GO:0008374"
#>  [76] "GO:0016972" "GO:0003857" "GO:0008320" "GO:0016462" "GO:0002161"
#>  [81] "GO:0004084" "GO:0003884" "GO:0004866" "GO:0000828" "GO:0019205"
#>  [86] "GO:0031267" "GO:0004029" "GO:0008318" "GO:0004731" "GO:0003867"
#>  [91] "GO:0017069" "GO:0004864" "GO:0030515" "GO:0004470" "GO:0004774"
#>  [96] "GO:0051723" "GO:0031543" "GO:0035596" "GO:0016289" "GO:0043175"
#> [101] "GO:0016415" "GO:0004058"
#>
#> $`L12 RN`
#> [1] "GO:0019829"
#>
#> $`L9 LN`
#>  [1] "GO:0030676" "GO:0008568" "GO:0005005" "GO:0005025" "GO:0009378"
#>  [6] "GO:0043138" "GO:0005247" "GO:0044020" "GO:0000980" "GO:0005313"
#> [11] "GO:0044748" "GO:0001162" "GO:0004705" "GO:0033192" "GO:0004439"
#> [16] "GO:0043813" "GO:0046975" "GO:0022841" "GO:0005228" "GO:0032143"
#> [21] "GO:0032142" "GO:0032181" "GO:0008186" "GO:0008311" "GO:0042800"
#> [26] "GO:0052629" "GO:0019799" "GO:0000979" "GO:0030899" "GO:0015333"
#> [31] "GO:0005248" "GO:0008510" "GO:0048101" "GO:0015184" "GO:0034639"
#> [36] "GO:0043139" "GO:0015279" "GO:0015141" "GO:0046976" "GO:0052907"
#> [41] "GO:0036402" "GO:0070615" "GO:0004535" "GO:1990189" "GO:1990190"
#> [46] "GO:0008310" "GO:0060002" "GO:0031151" "GO:0004176" "GO:0046974"
#> [51] "GO:0015181" "GO:0015189" "GO:0015093" "GO:0072320" "GO:0015183"
#> [56] "GO:0008273" "GO:0001006" "GO:0005415" "GO:0001003" "GO:0034597"
#> [61] "GO:0005366" "GO:0052909" "GO:0042799" "GO:0018423" "GO:0015131"
#> [66] "GO:0052906" "GO:0004671" "GO:0000064" "GO:0001164" "GO:0016316"
#>
#> $`L3 RN`
#>  [1] "GO:0003676" "GO:0008092" "GO:0004888" "GO:0000981" "GO:0000149"
#>  [6] "GO:0019904" "GO:0046983" "GO:0042802" "GO:0019899" "GO:0003713"
#> [11] "GO:0004497" "GO:0031072" "GO:0051536" "GO:0016651" "GO:0016757"
#> [16] "GO:0016746" "GO:0051213" "GO:0016705" "GO:0050839" "GO:0042277"
#> [21] "GO:0016849" "GO:0016798" "GO:0003906" "GO:0016627" "GO:0019825"
#> [26] "GO:0019208" "GO:0003916" "GO:0016810" "GO:0019838" "GO:0048038"
#> [31] "GO:0031369" "GO:0016788" "GO:0051018" "GO:0016684" "GO:0019955"
#> [36] "GO:0016765" "GO:0032182" "GO:0016817" "GO:0016614" "GO:0016772"
#> [41] "GO:0030247" "GO:0016846" "GO:0016866" "GO:0001882" "GO:0016830"
#> [46] "GO:0016638" "GO:0015144" "GO:0043021" "GO:0005539" "GO:0016701"
#> [51] "GO:0019207"
#>
#> $`L10 RN`
#> [1] "GO:0004970" "GO:1904315" "GO:0042626" "GO:0015271" "GO:0010485"
#> [6] "GO:0015378" "GO:0015278"
#>
```

```
#> $`L9 JN`
#> [1] "GO:0005328" "GO:0005231" "GO:0015379"
```

```r
# RN GO-terms on level 1 can be access as follows
visRDAGMF(organism = "Caenorhabditis elegans", plot = FALSE)$"L1 RN"
```

```
#> [1] "GO:0005198" "GO:0003824" "GO:0005215" "GO:0140110"
```

```r
# JN GO-terms on level 9 can be access as follows
visRDAGMF(organism = "Caenorhabditis elegans", plot = FALSE)$"L9 JN"
```

```
#> [1] "GO:0005328" "GO:0005231" "GO:0015379"
```

```r
# LN GO-terms on level 14 can be access as follows
visRDAGMF(organism = "Caenorhabditis elegans", plot = FALSE)$"L14 LN"
```

```
#> [1] "GO:0046961" "GO:0008553" "GO:0005391"
```

```r
# Represent the molecular function GO-DAG for organism Caenorhabditis elegans
visRDAGMF(organism = "Caenorhabditis elegans", plot = TRUE)[["plot"]]
```

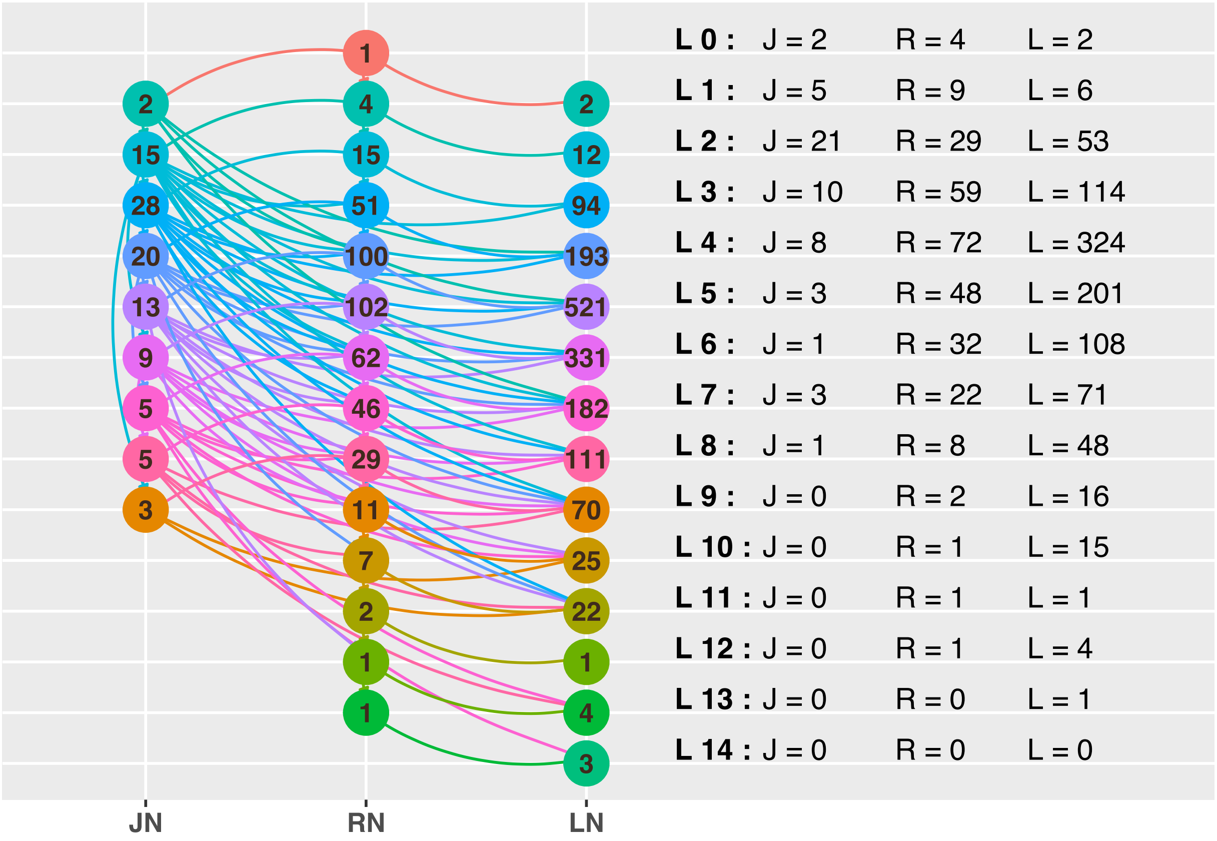

Figure 4: Visualization of a reduced GO-DAG for Caenorhabditis elegans.

visRsubDAGXX

The visRsubDAGXX function is similar to the visRDAGXX function, however, it visualizes an organism-specific sub-GO-DAG. The input of the function is a list of organism-specific GO-terms. If this list contains not all GO-terms of the organism, then category nodes are faded out. The XX in the function can be substituted with BP, MF or CC.

```r
Terms <- c("GO:0022403","GO:0000278","GO:0006414","GO:0006415","GO:0006614",
           "GO:0045047","GO:0072599","GO:0006613","GO:0000279","GO:0000087",
           "GO:0070972","GO:0000184","GO:0000280","GO:0007067","GO:0006413",
           "GO:0048285","GO:0006412","GO:0000956","GO:0006612","GO:0019080",
           "GO:0019083","GO:0016071","GO:0006402","GO:0043624","GO:0043241",
           "GO:0006401","GO:0072594","GO:0022904","GO:0019058","GO:0032984",
           "GO:0045333","GO:0006259","GO:0051301","GO:0022900","GO:0006396",
           "GO:0060337","GO:0071357","GO:0034340","GO:0002682","GO:0051320",
           "GO:0045087","GO:0051325","GO:0022411","GO:0016032","GO:0044764",
           "GO:0022415","GO:0051329","GO:0050776","GO:0030198","GO:0043062")

# visualisation the DAG node categories of the given biological process GO-terms
visRsubDAGBP(goterm = Terms, organism = "Human")
```

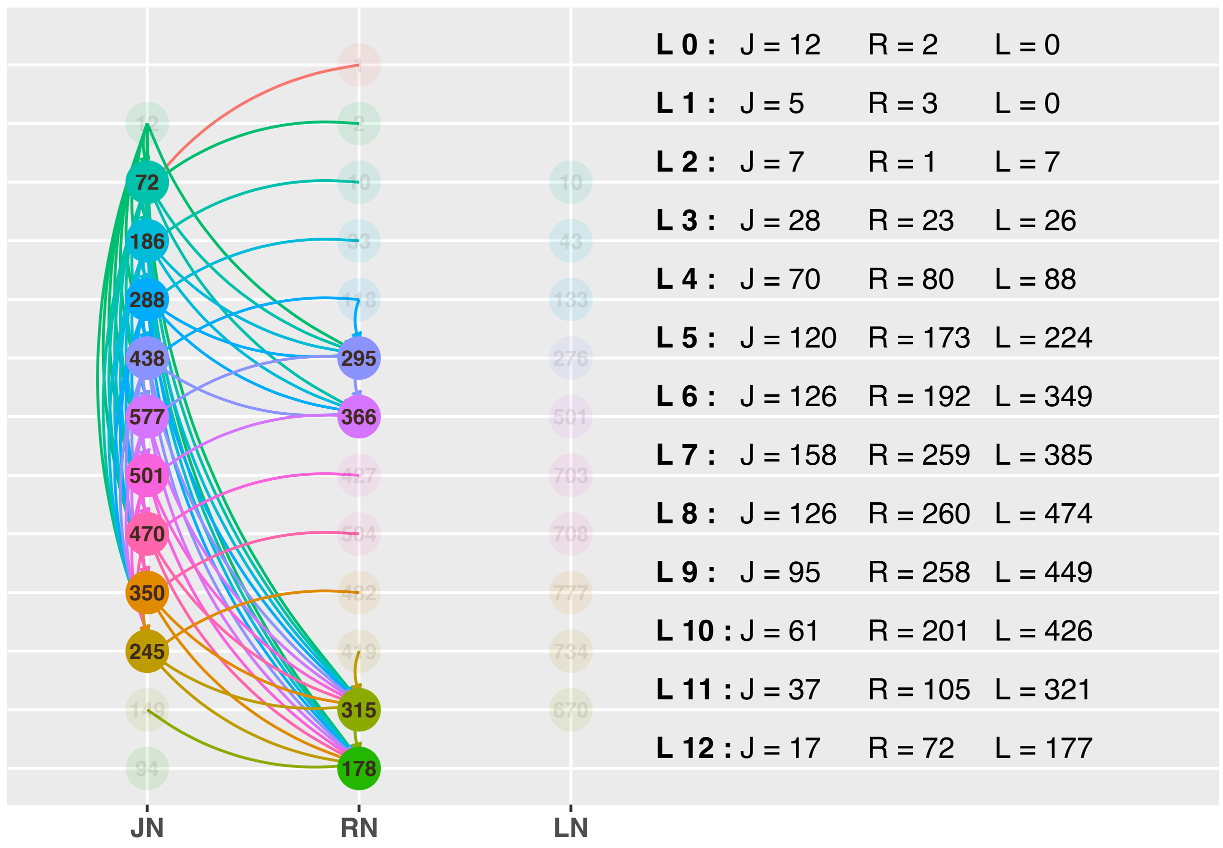

Figure 5: Visualization of a reduced sub-GO-DAG of BPs for Human

distRankingGO

Given a list of GO-terms, the function provides ranking for the GO-terms according to the distance between the GO-terms hierarchy level and the maximal depth of paths in the GO-DAG passing through these GO-terms. The function provides options for “BP”, “MF” and “CC” ontology.

```r
Terms <- c("GO:0000278","GO:0006414","GO:0022403","GO:0006415","GO:0006614",
           "GO:0045047","GO:0072599","GO:0006613","GO:0000184","GO:0070972",
           "GO:0006413","GO:0000087","GO:0000280","GO:0000279","GO:0006612",
           "GO:0000956","GO:0048285","GO:0019080","GO:0019083","GO:0043624",
           "GO:0006402","GO:0032984","GO:0006401","GO:0072594","GO:0019058",
```

```r
            "GO:0051301","GO:0016071","GO:0006412","GO:0002682","GO:0022411",
            "GO:0001775","GO:0046649","GO:0045321","GO:0050776","GO:0007155",
            "GO:0022610","GO:0060337","GO:0071357","GO:0034340","GO:0016032",
            "GO:0044764","GO:0006396","GO:0010564","GO:0002684","GO:0006259",
            "GO:0051249","GO:0045087")

# Ordering of the GO-terms in the list
distRankingGO(goterm = Terms, domain = "BP", plot = TRUE)
```

```
#> $`GO-terms_ranking`
#>  [1] "GO:0022610" "GO:0001775" "GO:0007155" "GO:0002682" "GO:0045321"
#>  [6] "GO:0000278" "GO:0051301" "GO:0046649" "GO:0050776" "GO:0002684"
#> [11] "GO:0048285" "GO:0044764" "GO:0000280" "GO:0022411" "GO:0010564"
#> [16] "GO:0051249" "GO:0032984" "GO:0016032" "GO:0045087" "GO:0070972"
#> [21] "GO:0043624" "GO:0006259" "GO:0016071" "GO:0034340" "GO:0006396"
#> [26] "GO:0019080" "GO:0006401" "GO:0072594" "GO:0019058" "GO:0006412"
#> [31] "GO:0071357" "GO:0072599" "GO:0006413" "GO:0019083" "GO:0006402"
#> [36] "GO:0060337" "GO:0006414" "GO:0022403" "GO:0000956" "GO:0045047"
#> [41] "GO:0000279" "GO:0006612" "GO:0006415" "GO:0006613" "GO:0000184"
#> [46] "GO:0000087" "GO:0006614"
#>
#> $indices_of_ranking
#>  [1] 36 31 35 29 33  1 26 32 34 44 17 41 13 30 43 46 22 40 47 10 20 45 27 39 42
#> [26] 18 23 24 25 28 38  7 11 19 21 37  2  3 16  6 14 15  4  8  9 12  5
#>
#> $distance
#>  [1] 17 16 16 15 15 14 14 14 14 14 13 13 12 12 12 12 11 11 11 10 10 10  8  8  8
#> [26]  7  7  7  7  7  7  6  6  6  6  6  4  4  4  3  3  3  2  2  2  2  1
#>
#> $plot
```

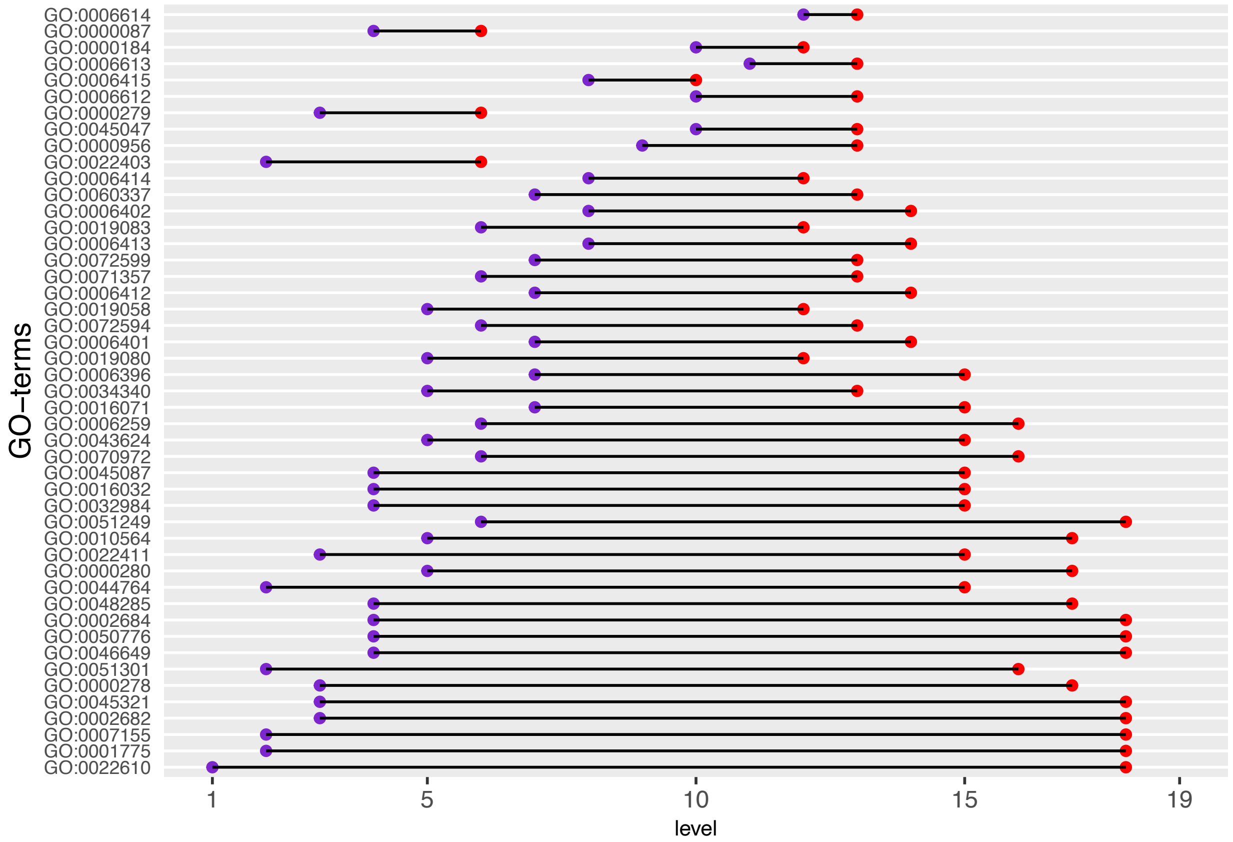

Figure 6: The hierarchy levels for a list of GO-terms (y-axis) are shown in purple and the hierarchy levels for the maximal depth of paths in the GO-DAG passing through these GO-terms is shown in red.

The function produced as output the ranked GO-terms, the indices of the ranking (indices corresponding to the original list), the distance between the GO-terms hierarchy level and the maximal depth of paths in the GO-DAG passing through these GO-terms and a visualisation of the ranking. The GO-terms are ranked according to the distance between the two points (purple and red) shown in Figure 6.

scoreRankingGO

The function scoreRankingGO is similar to the distRankingGO function because both function provide ordering for a given list of GO-terms. The difference is scoreRankingGO rank the GO-terms according to a score which is computed using Equation 1.

$$score = \frac{level(GO)}{level_{max}(GO)} \times \frac{level(GO)}{level_{GO-DAG}(GO)} \tag{1}$$

The function produced as output the ranked GO-terms, the indices of the ranking (indices corresponding to the original list), the scores of each GO-terms and a visualisation of the ranking.

```
Terms <- c("GO:0000278","GO:0006414","GO:0022403","GO:0006415","GO:0006614",
           "GO:0045047","GO:0072599","GO:0006613","GO:0000184","GO:0070972",
           "GO:0006413","GO:0000087","GO:0000280","GO:0000279","GO:0006612",
           "GO:0000956","GO:0048285","GO:0019080","GO:0019083","GO:0043624",
           "GO:0006402","GO:0032984","GO:0006401","GO:0072594","GO:0019058",
           "GO:0051301","GO:0016071","GO:0006412","GO:0002682","GO:0022411",
           "GO:0001775","GO:0046649","GO:0045321","GO:0050776","GO:0007155",
```

```
            "GO:0022610","GO:0060337","GO:0071357","GO:0034340","GO:0016032",
            "GO:0044764","GO:0006396","GO:0010564","GO:0002684","GO:0006259",
            "GO:0051249","GO:0045087")

# Ordering of the GO-terms in the list
scoreRankingGO(goterm = Terms, domain = "BP", plot = FALSE)
```

```
#> $GO_terms_ranking
#>  [1] "GO:0022610" "GO:0001775" "GO:0007155" "GO:0051301" "GO:0044764"
#>  [6] "GO:0002682" "GO:0045321" "GO:0000278" "GO:0022411" "GO:0022403"
#> [11] "GO:0046649" "GO:0050776" "GO:0002684" "GO:0048285" "GO:0032984"
#> [16] "GO:0016032" "GO:0045087" "GO:0000280" "GO:0010564" "GO:0000279"
#> [21] "GO:0043624" "GO:0034340" "GO:0051249" "GO:0019080" "GO:0019058"
#> [26] "GO:0070972" "GO:0006259" "GO:0000087" "GO:0072594" "GO:0071357"
#> [31] "GO:0019083" "GO:0016071" "GO:0006396" "GO:0006401" "GO:0006412"
#> [36] "GO:0072599" "GO:0060337" "GO:0006413" "GO:0006402" "GO:0006414"
#> [41] "GO:0000956" "GO:0006415" "GO:0045047" "GO:0006612" "GO:0000184"
#> [46] "GO:0006613" "GO:0006614"
#>
#> $indices_of_ranking
#>  [1] 36 31 35 26 41 29 33  1 30  3 32 34 44 17 22 40 47 13 43 14 20 39 46 18 25
#> [26] 10 45 12 24 38 19 27 42 23 28  7 37 11 21  2 16  4  6 15  9  8  5
#>
#> $score
#>  [1] 0.002923977 0.011695906 0.011695906 0.013157895 0.014035088 0.026315789
#>  [7] 0.026315789 0.027863777 0.031578947 0.035087719 0.046783626 0.046783626
#> [13] 0.046783626 0.049535604 0.056140351 0.056140351 0.056140351 0.077399381
#> [19] 0.077399381 0.078947368 0.087719298 0.101214575 0.105263158 0.109649123
#> [25] 0.109649123 0.118421053 0.118421053 0.140350877 0.145748988 0.145748988
#> [31] 0.157894737 0.171929825 0.171929825 0.184210526 0.184210526 0.198380567
#> [37] 0.198380567 0.240601504 0.240601504 0.280701754 0.327935223 0.336842105
#> [43] 0.404858300 0.404858300 0.438596491 0.489878543 0.582995951
```

prioritizedGOTerms

Given a vector of GO-terms, this function prioritizes the GO-terms by expoiting the structure of a DAG. Starting from the GO-term on the highest level and searching all the paths to the root node iteratively. If the argument "sp" is TRUE, only shortest paths are used, otherwise all paths. If any GO-terms in the input vector are found along this path, these GO-terms are removed. This is because the GO-term at the end of a path is more specific than the GO-terms along the path. For an organism, the GO-terms of that organism are used for the prioritization. If the organism argument is NULL then all the (non-retired) GO-terms from a particular ontology are used in the ranking.

```
Terms <- c("GO:0042254", "GO:0022613", "GO:0034470", "GO:0006364", "GO:0016072",
           "GO:0034660", "GO:0006412", "GO:0006396", "GO:0007005", "GO:0032543",
           "GO:0044085", "GO:0044281", "GO:0044257", "GO:0030163", "GO:0006082",
           "GO:0044248", "GO:0006519", "GO:0009056", "GO:0019752", "GO:0043436")

# We Prioritize the given biological process GO-terms

prioritizedGOTerms(lst = Terms, organism = "Human", sp = TRUE, domain = "BP")
```

```
#> $HF
#> [1] "GO:0006364" "GO:0032543" "GO:0044257" "GO:0019752" "GO:0007005"
#>
```

```
#> $rankHF
#> GO:0006364 GO:0032543 GO:0044257 GO:0019752 GO:0007005
#>          9          8          6          5          4
#>
#> $HI
#>  [1] "GO:0042254" "GO:0022613" "GO:0034470" "GO:0006364" "GO:0016072"
#>  [6] "GO:0034660" "GO:0006412" "GO:0006396" "GO:0007005" "GO:0032543"
#> [11] "GO:0044085" "GO:0044281" "GO:0044257" "GO:0030163" "GO:0006082"
#> [16] "GO:0044248" "GO:0006519" "GO:0009056" "GO:0019752" "GO:0043436"
#>
#> $rankHI
#> GO:0006364 GO:0034470 GO:0016072 GO:0032543 GO:0034660 GO:0006412 GO:0006396
#>          9          8          8          8          7          7          7
#> GO:0044257 GO:0030163 GO:0019752 GO:0042254 GO:0007005 GO:0043436 GO:0022613
#>          6          5          5          4          4          4          3
#> GO:0006082 GO:0044248 GO:0044085 GO:0044281 GO:0009056
#>          3          3          2          2          2
#>
#> attr(,"class")
#> [1] "GOxploreR" "GPrior"
```

GO4Organism

This function gives all the GO-terms association with an organism and their corresponding GO-levels.

```
# All the biological process gene association GO-terms for Human and their GO-level
head(GO4Organism(organism = "Human", domain = "BP"))
```

```
#>        GO ID Level
#> 1 GO:0006805     5
#> 2 GO:0042981     6
#> 3 GO:0006915     4
#> 4 GO:0043065     7
#> 5 GO:0031334     6
#> 6 GO:0090200     9
```

```
# All the molecular function gene association GO-terms for Mouse and their GO-level
head(GO4Organism(organism = "Mouse", domain = "MF"))
```

```
#>        GO ID Level
#> 1 GO:0005524     8
#> 2 GO:0004672     5
#> 3 GO:0004679     7
#> 4 GO:0016740     2
#> 5 GO:0000166     4
#> 6 GO:0016301     4
```

```
# All the cellular component gene association GO-terms for Rat and their GO-level
head(GO4Organism(organism = "Rat", domain = "CC"))
```

```
#>        GO ID Level
#> 1 GO:0016020     1
#> 2 GO:0016021     4
#> 3 GO:0005654    10
#> 4 GO:0005737     5
#> 5 GO:0005886     4
#> 6 GO:0005887     7
```

Conclusion

This vignette gave a brief overview of the functionality provided by the GOxploreR package. We showed all functions and how to use them.

References

1. Gene Ontology Consortium. 2018. The gene ontology resource: 20 years and still going strong. *Nucleic acids research* 47, D1: D330–D338.
